# Association between gut microbiota characteristics and therapeutic efficacy of multimodal combination therapy in hepatocellular carcinoma with portal vein tumor thrombus: a secondary analysis of a prospective phase II trial

**DOI:** 10.3389/fimmu.2026.1864941

**Published:** 2026-08-18

**Authors:** Xinwen Liang, Ye Li, Minhui Liang, Zhiming Zeng, Kai Hu, Lihua Yang, Hui Yan, Muyun Li, Jie Zeng, Jing Tang, Yufeng Lv, Li Liang, Jiali Meng, Huabin Su, Jie Ma, Ning Mo

**Affiliations:** 1 First Clinical Medical College, Guangxi Medical University, Nanning, Guangxi, China; 2 Department of Urology, The First Affiliated Hospital of Guangxi Medical University, Nanning, Guangxi, China; 3 Department of Oncology, The First Affiliated Hospital of Guangxi Medical University, Nanning, Guangxi, China; 4 Department of Gastroenterology, The First Affiliated Hospital of Guangxi Medical University, Nanning, Guangxi, China; 5 Department of Radiation Oncology, The First Affiliated Hospital of Guangxi Medical University, Nanning, Guangxi, China; 6 Department of Oncology, Foresea Life Insurance Guangxi Hospital, Nanning, Guangxi, China; 7 Department of Oncology, The Second Affiliated Hospital of Guangxi Medical University, Nanning, Guangxi, China; 8 Guangxi Key Laboratory of Immunology and Metabolism for Liver Diseases, Nanning, Guangxi, China; 9 Department of Science and Education, Jiangbin Hospital of Guangxi Zhuang Autonomous Region, Nanning, Guangxi, China

**Keywords:** cadonilimab, gut microbiota, hepatocellular carcinoma, immunotherapy, lenvatinib, portal vein tumor thrombus, stereotactic body radiotherapy

## Abstract

**Background:**

Hepatocellular carcinoma (HCC) with portal vein tumor thrombus (PVTT) carries a poor prognosis. Although SBRT combined with cadonilimab and lenvatinib has shown encouraging antitumor activity in a prior prospective phase II trial, marked interpatient heterogeneity remains. Gut microbiota may influence antitumor immunity and could help identify patients more or less likely to benefit from this regimen.

**Objective:**

To characterize baseline and longitudinal gut microbiota features in treatment-naïve HCC patients with PVTT receiving SBRT plus cadonilimab and lenvatinib, and to explore microbial signatures associated with therapeutic response.

**Methods:**

This is a complementary exploratory study nested within the aforementioned phase II trial. In this prospective multicenter exploratory cohort, 23 patients from the parent trial provided 46 paired fecal samples collected before treatment (Phase A) and after 3 cycles of cadonilimab (Phase B). Patients were categorized by best mRECIST response as responders (group C, CR/PR; n = 10) or non-responders (group D, SD/PD; n = 13), consistent with the parent trial’s grouping strategy. 16S rRNA amplicon sequencing and downstream bioinformatic analyses were performed to assess microbial diversity, differential taxa, predicted KEGG functional profiles, and exploratory discriminatory performance.

**Results:**

A total of 1,410,203 high-quality non-chimeric reads were retained and rarefied to 5,459 reads per sample. Baseline α-diversity indices were largely comparable between groups, although Faith’s phylogenetic diversity was higher in responders (P = 0.0178). β-diversity did not show clear between-group separation at baseline or marked restructuring after treatment. Genus-level DESeq2 analysis identified 7 baseline differential genera. *Extibacter* was absent in responders but detectable in 6/13 non-responders and showed the strongest discriminatory signal (P = 0.0179). Predicted functional analysis suggested differences in KEGG level 2 modules, and *[Ruminococcus]_gnavus_*group was positively correlated with “immune diseases” and “carbohydrate metabolism” after BH correction (q = 0.042). In exploratory ROC analysis, baseline *Extibacter* yielded an AUC of 0.731, increasing to 0.777 when combined with AFP.

**Conclusions:**

In HCC patients with PVTT treated with SBRT plus cadonilimab and lenvatinib, the overall gut microbiota architecture remained relatively stable, whereas selected baseline genus-level features were associated with therapeutic response. *Extibacter* showed an exploratory non-response-associated signal that requires validation in larger independent studies.

**Clinical Trial Registration:**

ClinicalTrials.gov, identifier NCT06040177.

## Introduction

Hepatocellular carcinoma (HCC) remains one of the most burdensome malignancies globally, with a particularly high prevalence in China (1–4). Despite continuous advances in the diagnostic and therapeutic landscape in recent years, the overall prognosis of advanced HCC remains dismal. The presence of portal vein tumor thrombosis (PVTT) is associated with more aggressive tumor biology, fewer therapeutic options, and elevated mortality risk, classifying it as one of the poorest-prognosis clinical subgroups in advanced HCC (5, 6).

The presence of PVTT not only signifies tumor invasion beyond local confines into major vascular structures but also markedly complicates subsequent therapeutic decision-making (5, 6). For this high-risk population, monotherapy with either local or systemic modalities rarely confers durable survival benefits. In recent years, amid the evolving paradigm of comprehensive HCC management, the treatment strategy for patients with PVTT has gradually shifted from traditional unimodal approaches toward individualized multidisciplinary integrated therapy (2–6). Against this backdrop, the combinatorial application of local and systemic treatments has garnered increasing attention. Particularly, the potential biological complementarity and clinical synergy among radiotherapy, immunotherapy, and targeted therapy offer novel therapeutic opportunities for this patient cohort (7–10).

Among local therapeutic modalities, stereotactic body radiotherapy (SBRT) has garnered increasing attention for HCC with PVTT in recent years, owing to its high precision, favorable dose conformity, and potent local tumor thrombus control capability (9, 10). Concurrently, systemic regimens typified by immune checkpoint inhibitors plus anti-angiogenic therapy have revolutionized the first-line treatment paradigm for unresectable HCC (7, 8). Notably, our team recently reported a prospective multicenter phase II trial that demonstrated SBRT combined with cadonilimab and lenvatinib elicits clinically meaningful antitumor activity and manageable toxicity in Vp3/Vp4 PVTT patients, supporting the feasibility and promise of this radiotherapy-immunotherapy-targeted triple strategy in this ultra-high-risk cohort (11). Nevertheless, marked heterogeneity in short-term response and disease control persists even under an identical treatment regimen, implying that clinical parameters alone are inadequate for accurate patient stratification.

Against this backdrop, the gut microbiota, a candidate biomarker with both biological relevance and clinical accessibility, has garnered mounting attention. Preclinical and translational evidence indicates that gut microecology modulates tumorigenesis and immunotherapy responses via diverse pathways, including bile acid metabolism, short-chain fatty acid synthesis, intestinal barrier homeostasis, and remodeling of innate and adaptive immunity (12–14). In HCC, the “hepatic-gut axis” represents an especially intimate connection: gut-derived metabolites, inflammatory signals, and microbiota-associated molecules directly contribute to shaping the liver immune microenvironment, rendering the gut microbiota a potential stratification marker (15–24).

Prior investigations in HCC and other hepatobiliary malignancies have demonstrated that baseline gut microbiota richness, specific taxa, microbiota-associated metabolites, and on-treatment longitudinal dynamics may correlate with anti-PD-1/PD-L1 therapeutic benefits, prognosis, or toxicity (15–21). Recent multi-omics studies have further revealed that integrated profiles of gut bacteria, fungi, and their metabolites may furnish more comprehensive information for evaluating immune checkpoint inhibitor efficacy (18–24). However, most existing studies have focused on systemic therapy alone or non-PVTT cohorts. For the high-risk subgroup of HCC with PVTT, systematic prospective evidence regarding baseline microbiome features, post-treatment longitudinal succession, and their associations with therapeutic outcomes within the SBRT-immunotherapy-targeted triple regimen remains scarce (11, 15–24).Building on our aforementioned phase II trial, the present study aimed to fill this gap by exploring the gut microbiota signatures of the same patient cohort, providing a microecological perspective for explaining the observed treatment response heterogeneity.

Against this background, this study prospectively enrolled treatment-naïve HCC patients with PVTT who received combined SBRT, cadonilimab, and lenvatinib. Paired pre- and post-treatment fecal samples were analyzed by 16S rRNA amplicon sequencing. We sought to address three key questions: first, whether baseline gut microbiota harbors specific signatures associated with short-term therapeutic response; second, whether the triple intervention elicits pronounced microbial community remodeling; third, whether core differential taxa integrated with functional inference and clinical parameters can yield exploratory predictive clues for treatment efficacy (11, 15–24).

## 2 Materials and methods

### 2.1 Study design and study subjects

**Figure 1:**
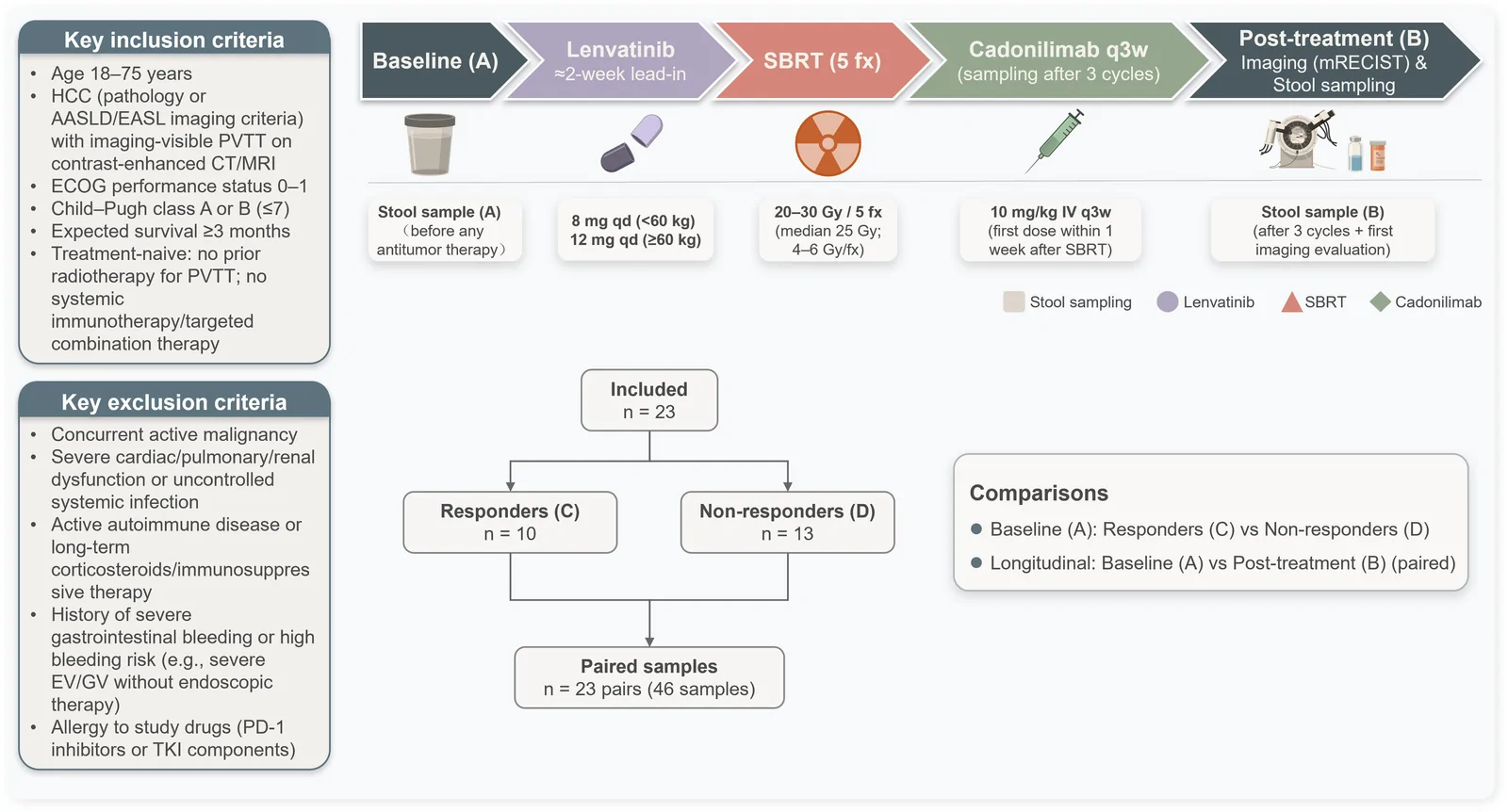
Research design, treatment protocol and biological sample collection process. Flowchart of patient enrollment and grouping. A total of 23 HCC patients complicated by PVTT who received SBRT plus cadonilimab and lenvatinib were enrolled. Patients were classified into responders (Group C: CR + PR, n = 10) and non-responders (Group D: SD + PD, n = 13) based on best objective response per mRECIST. Paired fecal samples were collected at baseline (before treatment, Phase A) and at the first efficacy evaluation after 3 cycles of cadonilimab (Phase B), yielding 46 samples for 16S rRNA amplicon sequencing. Treatment and sampling timeline. Patients received induction lenvatinib followed by SBRT, and cadonilimab maintenance was initiated within 1 week after radiotherapy. Arrows indicate bioinformatic comparisons: baseline C vs D, pre- vs post-treatment longitudinal changes, and post-treatment C vs D. Abbreviations: HCC, hepatocellular carcinoma; PVTT, portal vein tumor thrombus; SBRT, stereotactic body radiation therapy; CR, complete response; PR, partial response; SD, stable disease; PD, progressive disease; q3w, every 3 weeks.

This is a complementary exploratory study nested within the prospective multicenter phase II trial NCT06040177; Mo N et al., 2025 (11).This study employed a prospective, multicenter, exploratory cohort design and was conducted between February and December 2023 across three institutions: the First Affiliated Hospital of Guangxi Medical University, the Second Affiliated Hospital of Guangxi Medical University, and Foresea Life Insurance Guangxi Hospital. A total of 23 treatment-naïve patients with hepatocellular carcinoma (HCC) and portal vein tumor thrombus (PVTT) were enrolled. All patients met the clinical diagnostic criteria outlined in *the Guidelines for the Diagnosis and Treatment of Primary Liver Cancer* or had a confirmed pathological diagnosis (**Figure 1**), with inclusion and exclusion criteria identical to those described in the parent trial.

The parent trial eligibility criteria, treatment protocol, sample collection procedures, and clinical assessment framework were consistent with those of the parent study. Several major clinical exposures that could affect microbiota profiles were partially restricted by the parent trial eligibility criteria, including active infection requiring systemic anti-infective treatment shortly before treatment initiation and systemic corticosteroid or immunosuppressive therapy. Other microbiome-specific concomitant exposures were not predefined variables in the current microbiome-specific analytic framework and were therefore considered in interpretation rather than included in formal multivariable adjustment.

The study protocol was reviewed and approved by the Ethics Committee of the First Affiliated Hospital of Guangxi Medical University (Approval No. 2022-K139-01) and was conducted in accordance with the Declaration of Helsinki. Written informed consent was obtained from all participants prior to enrollment.

### 2.2 Treatment protocol

Enrolled patients initially received induction lenvatinib, followed by sequential stereotactic body radiotherapy (SBRT; median prescribed dose: 25 Gy/5 fx). Cadonilimab (10 mg/kg, q3w) maintenance therapy was initiated within 1 week after SBRT completion, establishing a multimodal SBRT-immunotherapy-targeted therapy regimen.

### 2.3 Efficacy assessment and grouping definition

Clinical efficacy was dynamically assessed per the modified Response Evaluation Criteria in Solid Tumors (mRECIST), with best overall response serving as the stratification endpoint. Patients achieving complete response (CR) or partial response (PR) were defined as the response group, whereas those with stable disease (SD) or progressive disease (PD) were categorized as the non-response group.

### 2.4 Sample collection, storage and transportation

Paired fecal samples were collected from patients prior to any antitumor intervention (baseline, Phase A) and at the first radiologic assessment following completion of 3 cycles of cadonilimab therapy (Phase B). Following standardized SOPs, samples were immediately snap-frozen in liquid nitrogen for 15 minutes to quench metabolic activity, then transferred to a −80 °C ultra-low-temperature freezer for storage. All samples were shipped to the testing center under dry ice cold-chain conditions for nucleic acid extraction and amplicon sequencing.

### 2.5 16S rRNA amplicon sequencing

Total genomic DNA was extracted from fecal samples using the CTAB method. After quality assessment via 1% agarose gel electrophoresis, DNA was diluted to 1 ng/μL. The V3–V4 hypervariable regions of the 16S rRNA gene were amplified by PCR using the 341F/806R primer set. Amplicons were purified with a Universal DNA Purification Kit, and sequencing libraries were constructed using the NEB Next^®^ Ultra DNA Library Prep Kit. Following quality validation on the Agilent 5400, libraries were subjected to 250 bp paired-end (PE250) sequencing on the Illumina platform. Per-sample sequencing depth and DADA2 read-retention statistics are provided in **Supplementary Table 1**.

### 2.6 Bioinformatics processing and statistical analysis

Raw FASTQ reads were imported into QIIME2 (25). The DADA2 pipeline was used for denoising, paired-end merging and chimera removal to construct an amplicon sequence variant (ASV) table (26). Taxonomic classification was completed against the SILVA 138.2 16S rRNA database in QIIME2, with mitochondrial and chloroplast sequences discarded (27). Owing to limited species-level resolution of V3–V4 16S rRNA sequencing, subsequent analyses focused primarily on genus-level taxonomy; available species-level annotations were only interpreted exploratorily.

To mitigate bias caused by unequal sequencing depth, all samples were rarefied to an equal depth of 5,459 reads prior to α- and β-diversity analysis. This cutoff matched the minimum valid read count post-DADA2 filtering across all samples, preserving all 46 paired fecal samples for diversity profiling. Sample-wise read retention metrics from DADA2 are listed in **Supplementary Table 1**. A sensitivity analysis excluding the shallowest-depth sample A05 was conducted to evaluate whether the baseline diversity and Extibacter patterns were driven by the lowest-depth sample, with results summarized in **Supplementary Table 10**.

All α- and β-diversity calculations were implemented in QIIME2 (version 2022.2). Differential taxa were screened using DESeq2 (v1.26.0) and LEfSe (v1.0.8) (28, 29). Community functional capacity was predicted from 16S data, and functional discrepancies were compared at the KEGG level 2 pathway hierarchy (30). Spearman rank correlation was applied to build co-occurrence networks connecting differential genera, predicted functional modules and clinical phenotypes. Exploratory ROC curves were generated to test whether baseline *Extibacter*, individually or combined with AFP, could distinguish treatment non-response; 1,000 bootstrap resampling cycles were applied to internally assess the stability of both models. These ROC analyses remained exploratory rather than formal external validation due to the modest sample size. Intergroup comparisons were implemented via Student’s t-test, Mann–Whitney U test, chi-squared test or Fisher’s exact test. The Benjamini–Hochberg (BH) method was adopted for multiple testing correction, with statistical significance defined as *P* < 0.05 or *q* < 0.05.

## 3 Results

### 3.1 Clinical cohort baseline characteristics and efficacy assessment

This microbiome cohort was derived from the parent phase II trial with identical eligibility criteria, treatment regimens and clinical evaluation framework. A total of 23 HCC patients complicated with PVTT were prospectively enrolled, and 46 paired fecal samples were successfully acquired for downstream microbiome profiling. Based on mRECIST best overall response stratification, 10 patients were classified into the response group (Group C) and 13 into the non-response group (Group D). Core clinical variables including tumor burden, baseline alpha-fetoprotein (AFP) level, liver function grade, and viral infection status were well balanced between groups, yielding highly comparable baseline characteristics (**Table 1**; only the ECOG score exhibited a marginal difference, P = 0.041).

**Table 1:** Baseline clinical characteristics of patients with HCC and PVTT according to treatment response.

Variable	Level	Overall	C group	D group	P value
Tumor volume		0.30 (0.20, 0.41)	0.27 (0.19, 0.47)	0.35 (0.25, 0.41)	1.000
Largest intrahepatic lesion diameter, cm		11.00 (7.50, 14.50)	8.70 (6.33, 11.05)	13.00 (10.20, 15.00)	0.145
Baseline AFP		2906.00 (74.18, 9274.50)	2501.00 (17.83, 8785.50)	2906.00 (135.00, 12450.00)	0.515
Baseline DCP		17162.00 (5812.00, 43676.50)	7909.00 (3441.00, 20942.75)	23180.00 (7087.00, 53041.00)	0.129
Age, years		53.00 (45.50, 58.50)	52.50 (45.25, 58.75)	53.00 (47.00, 56.00)	1.000
ECOG performance status		1.00 (0.00, 1.00)	0.00 (0.00, 1.00)	1.00 (1.00, 1.00)	0.041
VP classification	VP3	12 (52.2%)	6 (60.0%)	6 (46.2%)	0.812
	VP4	11 (47.8%)	4 (40.0%)	7 (53.8%)	
Cheng’s classification	Type II	12 (52.2%)	6 (60.0%)	6 (46.2%)	0.619
	Type III	7 (30.4%)	3 (30.0%)	4 (30.8%)	
	Type IV	2 (8.7%)	0 (0.0%)	2 (15.4%)	
	Inferior vena cava tumor thrombus	2 (8.7%)	1 (10.0%)	1 (7.7%)	
Cirrhosis	No	4 (17.4%)	2 (20.0%)	2 (15.4%)	1.000
	Yes	19 (82.6%)	8 (80.0%)	11 (84.6%)	
Viral infection	HBV	22 (95.7%)	10 (100.0%)	12 (92.3%)	1.000
	HBV+HCV	1 (4.3%)	0 (0.0%)	1 (7.7%)	
Child–Pugh class	A	21 (91.3%)	8 (80.0%)	13 (100.0%)	0.347
	B	2 (8.7%)	2 (20.0%)	0 (0.0%)	
Metastatic site	Liver	12 (52.2%)	6 (60.0%)	6 (46.2%)	0.619
	Liver + lung	2 (8.7%)	1 (10.0%)	1 (7.7%)	
	Liver + lymph node	7 (30.4%)	3 (30.0%)	4 (30.8%)	
	Liver + abdominopelvic cavity	2 (8.7%)	0 (0.0%)	2 (15.4%)	
Number of tumor lesions	A (1 lesion)	5 (21.7%)	3 (30.0%)	2 (15.4%)	0.424
	B (2–5 lesions)	13 (56.5%)	6 (60.0%)	7 (53.8%)	
	C (>5 lesions)	5 (21.7%)	1 (10.0%)	4 (30.8%)	

Data are presented as median (P25, P75) or n (%). Responders (group C: CR/PR) and non-responders (group D: SD/PD) were defined by mRECIST. PVTT: portal vein tumor thrombus; HBV: hepatitis B virus; HCV: hepatitis C virus; AFP: alpha-fetoprotein; DCP: des-gamma-carboxy prothrombin; ECOG: Eastern Cooperative Oncology Group. DCP values reported as >300000 were truncated to 300000 for statistical analysis.

### 3.2 High-throughput quality control for amplicon sequencing data

Raw sequencing generated 4,628,972 reads across 46 fecal samples. After quality filtering, DADA2 denoising, paired-end merging, and chimera removal, 1,410,203 high-quality non-chimeric reads were retained, with a median of 29,428.5 reads per sample (IQR: 19,683.25–41,553.75). The minimum number of non-chimeric reads was 5,459; therefore, an even rarefaction depth of 5,459 reads per sample was used for α- and β-diversity analyses to retain all paired samples. Rarefaction curves approached saturation at this cutoff, supporting the adequacy of sequencing depth for subsequent diversity exploration. Detailed per-sample DADA2 read-retention statistics are summarized in **Supplementary Table 1**.

To further assess whether the relatively low rarefaction depth influenced the main baseline findings, we conducted a sensitivity analysis after excluding A05, the lowest-depth sample. Overall α-diversity patterns remained broadly consistent with primary analysis, and Bray–Curtis-based PERMANOVA still detected no significant intergroup community separation. After removing A05, *Extibacter* remained undetectable in treatment responders, while it was still recovered in 6 out of 13 non-responder samples. These observations suggest that the main exploratory baseline findings were not solely driven by this lowest-depth sample (**Supplementary Table 10**).

### 3.3 Diversity landscape of the baseline gut microbiota

**Figure 2:**
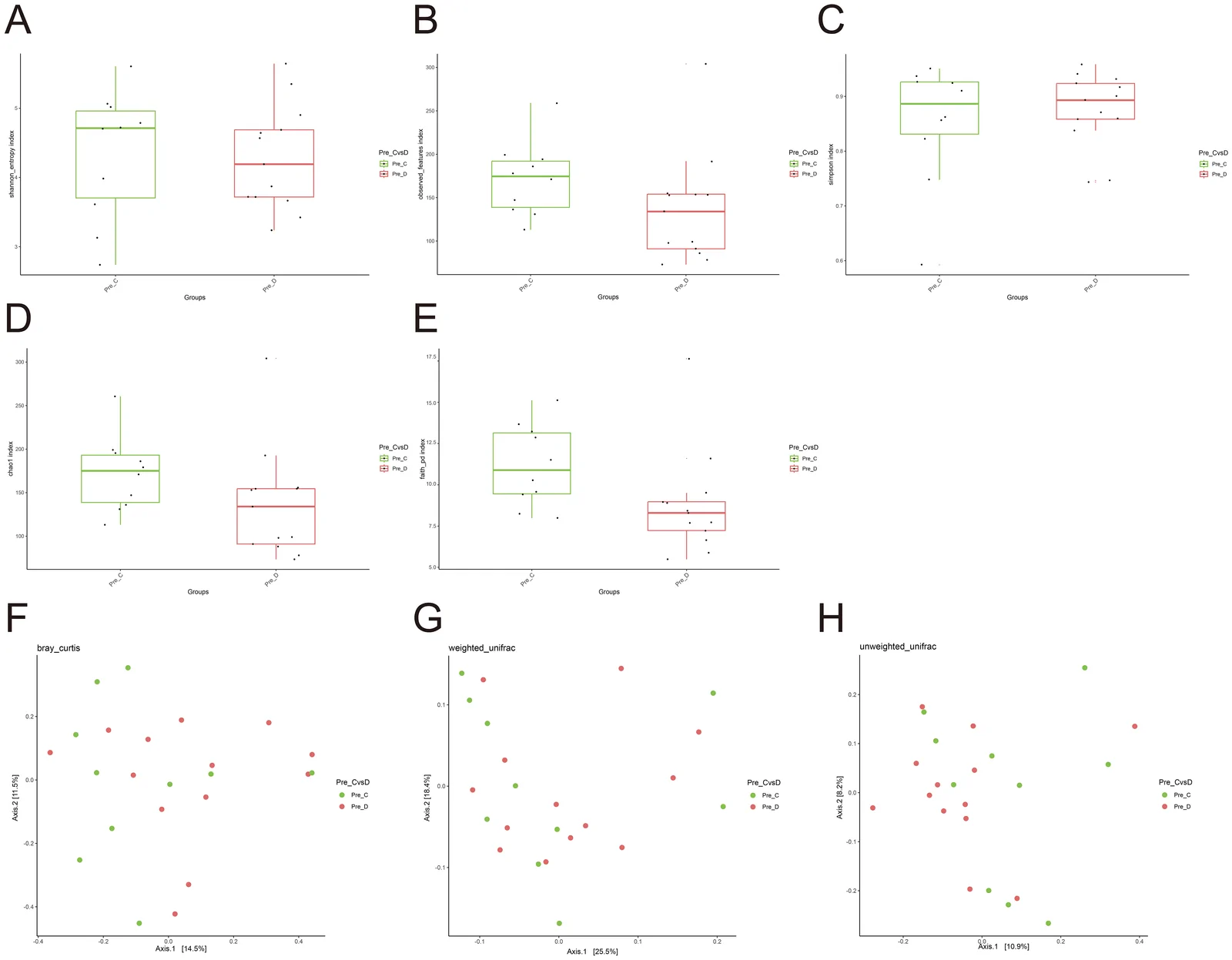
Comparison of α/β diversity of gut microbiota at baseline. **(A)** Shannon index boxplot; **(B)** Observed features boxplot; **(C)** Simpson index boxplot; **(D)** Chao1 boxplot; **(E)** Faith’s PD boxplot; **(F)** PCoA based on Bray–Curtis distance; **(G)** PCoA based on weighted UniFrac distance; **(H)** PCoA based on unweighted UniFrac distance. Boxplots compare baseline α-diversity indices between responders (Group C, n = 10) and non-responders (Group D, n = 13). Faith’s PD was significantly higher in responders (P = 0.0178), whereas no significant differences were observed for other indices. No distinct separation was observed in PCoA using any of the three distance metrics.

Baseline α-diversity analysis revealed no significant between-group differences in the Shannon, Observed features, Simpson, or Chao1 indices; only Faith’s PD was significantly higher in the response group (P = 0.0178), indicating broader phylogenetic diversity coverage in treatment-sensitive patients (**Figures 2A–E**; **Supplementary Table 2a**). Consistent with this, β-diversity analysis showed no robust separation between groups. PCoA based on Bray–Curtis, weighted UniFrac, and unweighted UniFrac distances revealed no distinct clustering (**Figures 2F–H**), and PERMANOVA results were nonsignificant (Bray–Curtis: q = 0.586; weighted UniFrac: q = 0.572; unweighted UniFrac: q = 0.514; **Supplementary Table 3**), implying minimal divergence in overall community structure at baseline. Additional baseline β-diversity visualizations are shown in **Supplementary Figure 5**.

**Figure 3:**
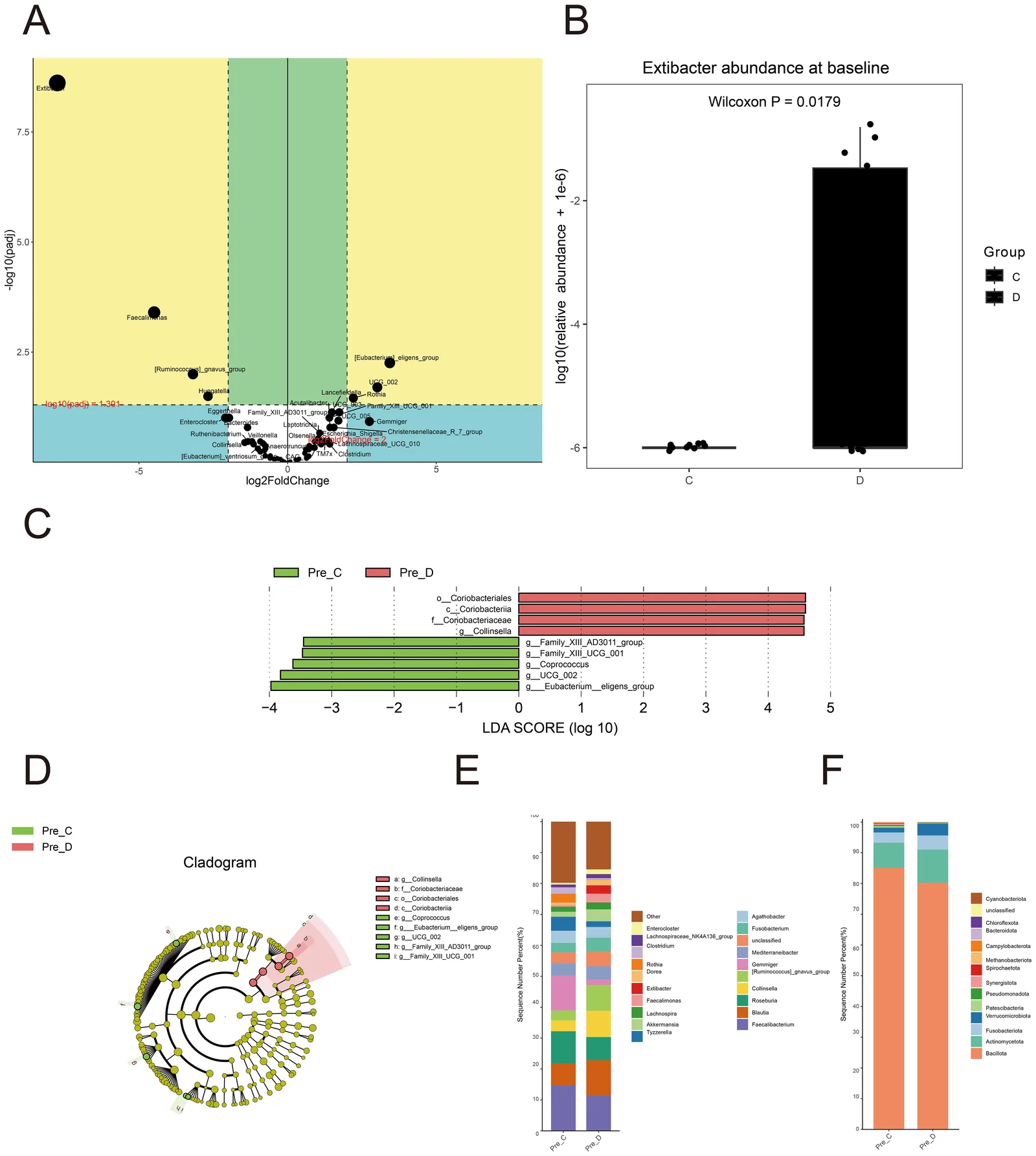
Analysis of efficacy-related differential bacterial genera at baseline. **(A)** DESeq2 volcano plot at genus level; **(B)** boxplot of baseline relative abundance of *Extibacter*; **(C)** LEfSe LDA score plot at genus level; **(D)** LEfSe phylogenetic cladogram at genus level; **(E)**, community composition plot at genus level; **(F)**, community composition plot at phylum level. The volcano plot identified 7 differential genera with padj < 0.05. *Extibacter*, *Faecalimonas*, *[Ruminococcus]_gnavus*_group, and *Hungatella* were significantly enriched in non-responders, whereas [*Eubacterium]_eligens_group*, UCG_002, and *Rothia* were enriched in responders.

At the community composition level, *Bacillota* was the most dominant phylum in baseline samples, followed by *Actinomycetota* and *Fusobacteriota*. The predominant genera included *Faecalibacterium*, *Roseburia*, *Blautia*, *[Ruminococcus]_gnavus*_group, *Agathobacter*, *Collinsella*, and *Akkermansia*. Integrating compositional profiles and differential abundance analyses, the response group was enriched for signals associated with *Rothia*, UCG_002, and *Akkermansia*, whereas the non-response group exhibited elevated abundances of *Extibacter*, *Faecalimonas*, and [*Ruminococcus]_gnavus*_group (**Figures 3E, F**; **Supplementary Figures 3A, B**).

### 3.4 Genus-level differential microbiota features associated with therapeutic response at baseline

Because V3–V4 16S rRNA sequencing has limited resolution at the species and strain levels, the main differential-abundance analysis and biological interpretation were conducted at the genus level. Although overall diversity and community structure exhibited modest differences at baseline, genus-level differential abundance analysis (DESeq2) identified seven significantly differential genera associated with therapeutic efficacy (padj < 0.05): *Extibacter*, *Faecalimonas*, [*Eubacterium*]*_eligens_group*, [*Ruminococcus]_gnavus*_group, UCG_002, *Hungatella*, and *Rothia*. This suggests that efficacy-related signals were more concentrated in specific taxa than at the global community level (**Table 2**).

**Table 2:** Analysis of core differential bacterial genera between responders and non-responders at baseline (DESeq2).

Genus	baseMean	log2FoldChange	P value	Adjusted P value
*Extibacter*	150.87	-7.747	3.38×10^−^¹¹	2.43×10^−9^
*Faecalimonas*	147.01	-4.491	1.10×10^−5^	3.97×10^−4^
*[Eubacterium]_eligens_group*	62.15	3.434	2.32×10^−4^	5.57×10^−^³
*[Ruminococcus]_gnavus_group*	519.96	-3.187	5.58×10^−4^	1.00×10^−^²
*UCG_002*	38.80	3.015	1.39×10^−^³	1.99×10^−^²
*Hungatella*	22.82	-2.680	2.66×10^−^³	3.19×10^−^²
*Rothia*	19.64	2.208	3.40×10^−^³	3.50×10^−^²

Genera with adjusted P < 0.05 are shown. baseMean: normalized mean abundance; log2FoldChange > 0 indicates enrichment in responders, while < 0 indicates enrichment in non-responders; Adjusted P value: Benjamini-Hochberg corrected P value.

Of these, [*Eubacterium]_eligens_group*, UCG_002, and *Rothia* were enriched in the response group, whereas *Extibacter*, *Faecalimonas*, *[Ruminococcus]_gnavus*_group, and *Hungatella* were enriched in the non-response group (**Figure 3A**; **Table 2**). The corresponding log2 fold-change (log2FC) and adjusted P-values (padj) were as follows: [*Eubacterium]_eligens_group*, 3.434 and 0.0056; UCG_002, 3.015 and 0.0200; *Rothia*, 2.208 and 0.0350; *Extibacter*, − 7.747 and 2.43×10^−9^; *Faecalimonas*, − 4.491 and 3.97×10^−4^; *[Ruminococcus]_gnavus*_group, − 3.187 and 0.0100; *Hungatella*, − 2.680 and 0.0319.

Among them, *Extibacter* showed the most pronounced between-group distribution. Sample-level profiles showed that *Extibacter* was undetectable in all 10 baseline samples from the response group, whereas 6 of 13 samples in the non-response group exhibited non-zero abundance. The Wilcoxon rank-sum test showed a significant difference (P = 0.0179; **Figure 3B**; **Supplementary Table 7**). This pattern remained broadly consistent in sensitivity analysis excluding the lowest-depth sample A05 (**Supplementary Table 10**).

LEfSe analysis and phylum/genus-level compositional plots were largely consistent with DESeq2 results in directional trends, further supporting that efficacy-related differences were predominantly concentrated at the genus level. Supplementary taxonomic summaries at the phylum and family levels, together with exploratory species-level annotations, are presented in **Figure 3C–F**; **Supplementary Tables 5a–c**.

### 3.5 SBRT-immunotherapy-targeted combined intervention: longitudinal dynamic succession of intestinal microecology

To evaluate the longitudinal effects of combination therapy on the gut microbiota, we compared paired samples from 23 patients at baseline (Phase A) and after 3 cycles of cadonilimab (Phase B). Overall analysis revealed no significant changes in any α-diversity indices (**Supplementary Figure 4A**; **Supplementary Table 2b**). Following stratification, no significant alterations in α-diversity indices were observed in the response group (**Supplementary Figure 4B**; **Supplementary Table 2c**). In the non-response group, only observed features and chao1 exhibited an increasing trend, whereas Faith’s PD was not statistically significant (P = 0.125; **Supplementary Figure 4C**; **Supplementary Table 2d**). Overall paired β-diversity results are displayed in **Supplementary Figure 7A–C** and **Supplementary Table 8**; no significant separation in β-diversity was observed between groups after treatment (**Supplementary Figures 8A–C**; **Supplementary Table 9**).

### 3.6 16S-inferred functional profiles and exploratory genus–function associations at baseline

**Figure 4:**
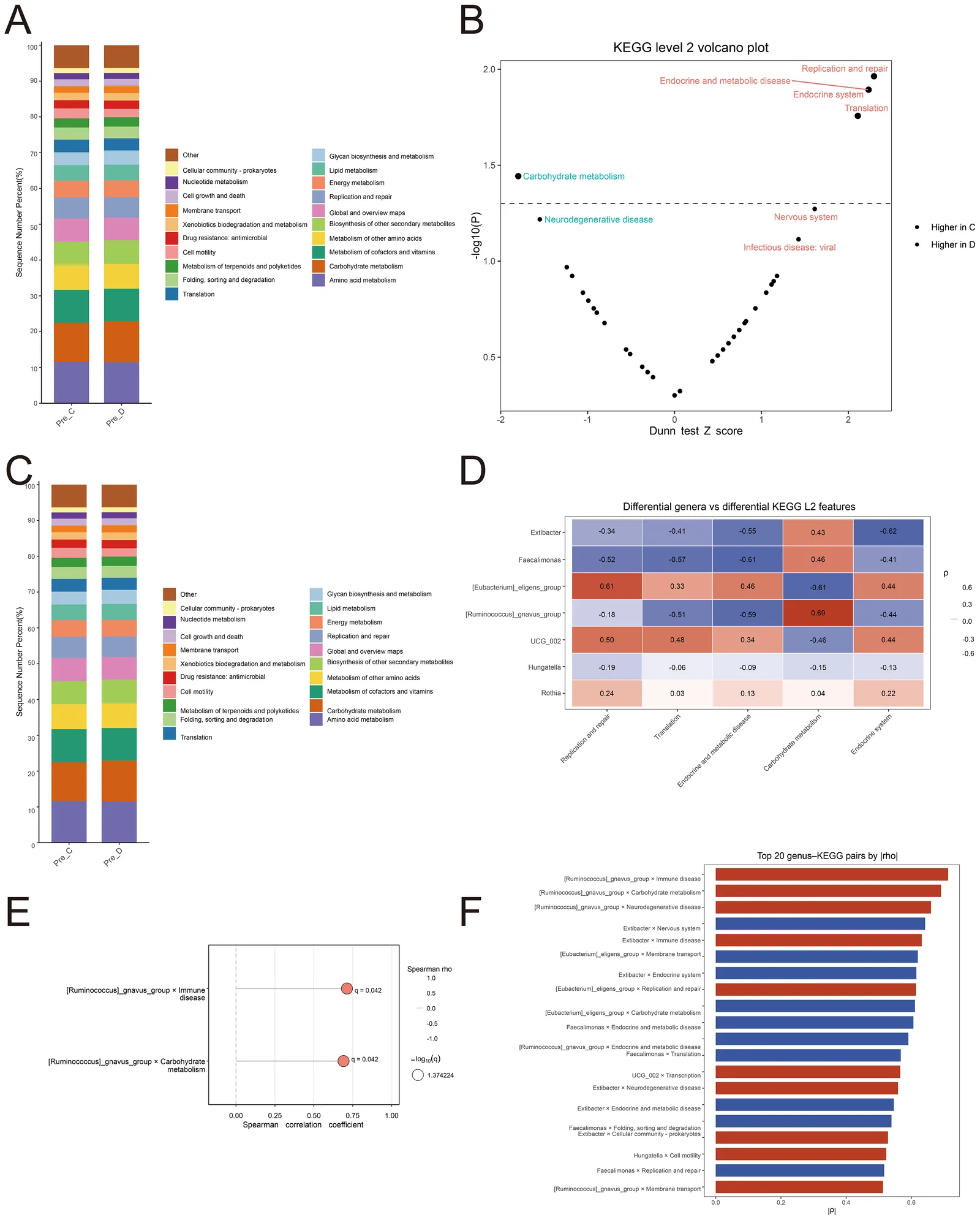
16S-inferred functional profiles and exploratory genus–function associations at baseline. **(A)** Community composition plot of KEGG L2 pathways; **(B)** Differential volcano plot of KEGG L2 pathways; **(C)** Visualization of dominant KEGG L2 modules; **(D)** Heatmap of correlations between differential genera and KEGG L2 modules; **(E)** Genus-function (genus–KEGG pair) associations remaining significant after BH correction; **(F)** Top 20 correlated feature pairs ranked by absolute Spearman’s rho (|rho|). BH, Benjamini-Hochberg.

**Figure 5:**
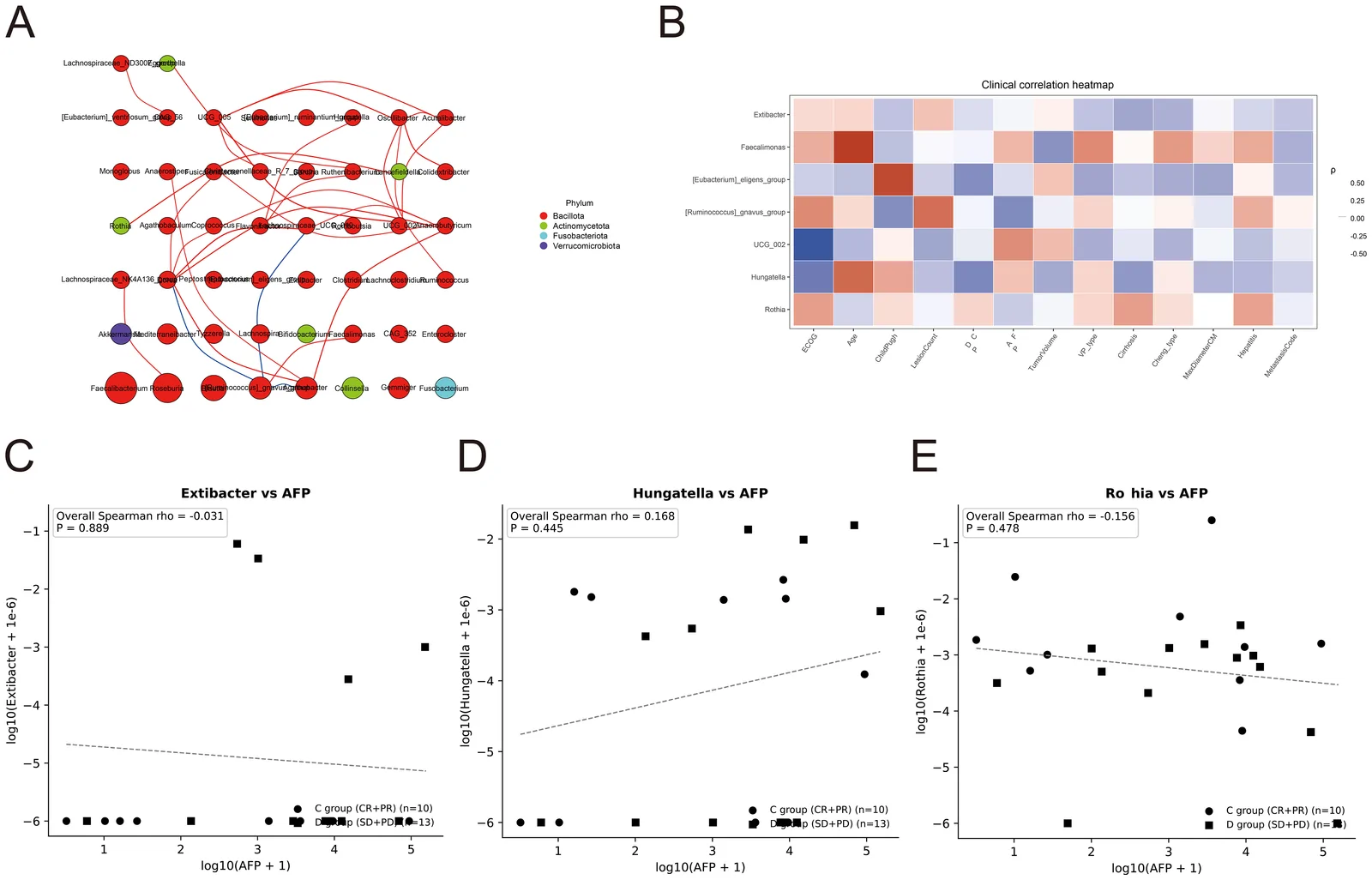
Exploratory network and clinical correlation analyses of differential genera. **(A)** Microbial interaction network; **(B)** Spearman correlation heatmap between differential genera and key clinical indicators; **(C)** Scatter plot showing the correlation between *Extibacter* and AFP; **(D)** Scatter plot showing the correlation between *Hungatella* and AFP; **(E)** Scatter plot showing the correlation between *Rothia* and AFP. These correlation analyses were exploratory and do not imply causality.

Functional potential inference based on 16S rRNA data revealed differential abundances in several KEGG L2 modules between the two groups (**Figures 4A–C**). Further Spearman rank correlation analysis with Benjamini–Hochberg correction demonstrated that [*Ruminococcus]_gnavus_group*, which was enriched in the non-response group, was significantly positively correlated with the “immune diseases” and “carbohydrate metabolism” modules (rho = 0.713 and 0.691; q = 0.042; **Figures 4D, E**). Microbial co-occurrence network analysis indicated that this taxon occupied a highly connected node (**Figure 5A**), but these network and functional correlations were derived from 16S-inferred profiles and do not demonstrate direct biological activity or causality. Detailed baseline KEGG level 2 results and genus–KEGG correlation statistics are provided in **Supplementary Figure 6**, **Supplementary Tables 4**, **6**.

#### 3.6.1 Interaction network of key differential genera, metabolism and immune modules

Following Spearman rank correlation analysis and Benjamini–Hochberg correction, no statistically significant associations were detected between the seven differentially abundant genera and key baseline clinical variables (**Figures 5B–E**). However, given the small sample size, sparse abundance patterns of several taxa, and absence of formal multivariable adjustment, these findings do not establish that the observed microbial features were independent of clinical characteristics.

#### 3.6.2 Exploratory discriminatory analysis based on core microbiota and AFP

**Figure 6:**
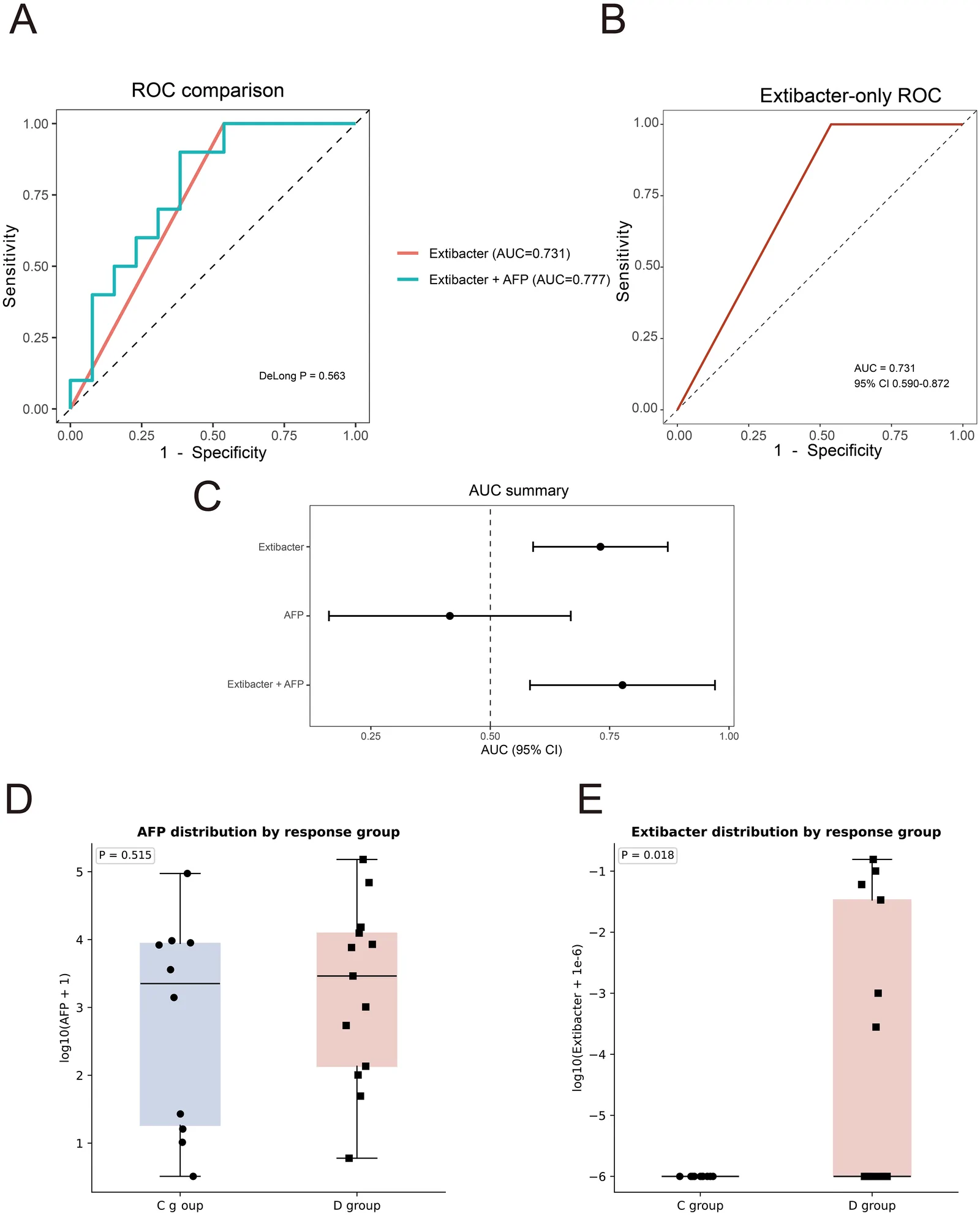
Exploratory discriminatory analysis based on Extibacter and AFP. **(A)** ROC curve comparison between the *Extibacter* single model and the *Extibacter* + AFP dual-variable combined model; **(B)** ROC curve of the *Extibacter* single model; **(C)** Summary plot of AUC and 95% CI for different models; **(D)** Distribution of AFP between the two groups; **(E)** Distribution of *Extibacter* between the two groups. The gray dotted line indicates the random discrimination reference line.

Building on this, we further evaluated the exploratory discriminatory value of *Extibacter* for treatment non-response. ROC curve analysis demonstrated that the area under the curve (AUC) of *Extibacter* alone in predicting treatment non-response was 0.731 (95% CI: 0.590–0.872; **Figure 6B**). When combined with baseline AFP levels, the AUC increased to 0.777 (95% CI: 0.583–0.971; **Figures 6A, C**). The distribution plots are shown in **Figures 6D, E**, and the apparent AUCs were estimated from the same small cohort. The Extibacter signal was assessed only exploratorily. Bootstrap-based internal assessment yielded mean AUCs of 0.729 for Extibacter alone and 0.801 for Extibacter plus AFP across 1,000 resampling iterations; however, this procedure assessed only internal stability and did not constitute external validation (**Supplementary Table 11**).

## 4 Discussion

Patients with HCC and PVTT frequently exhibit marked heterogeneity in treatment efficacy when receiving SBRT plus immunotherapy and targeted therapy (5, 6, 11). In this prospective longitudinal cohort, the overall gut microbiota structure showed no significant separation between response groups or marked longitudinal restructuring, whereas several genus-level taxa differed between groups. These results suggest that response-associated signals in this dataset were concentrated in selected taxa rather than in global community structure (12–24). Given the small cohort and exploratory design, these observations should be interpreted as hypothesis-generating associations rather than evidence of microbial determinants of treatment efficacy.

Inspection of α-diversity indices revealed that the response group exhibited higher Faith’s PD at baseline, indicating broader phylogenetic representation in responders. Previous investigations of immunotherapy in HCC or other hepatobiliary malignancies have reported associations between baseline microbiota diversity and treatment response (15, 17–24). The biological relevance of this isolated diversity difference remains uncertain, and the present data do not establish that higher phylogenetic diversity contributed to treatment response. Of note, longitudinal analysis demonstrated that only Observed features and Chao1 showed an increasing trend in the non-response group, whereas Faith’s PD did not attain statistical significance (P = 0.125). These fluctuations are best interpreted as descriptive ecological observations rather than evidence of a unidirectional beneficial effect.

In contrast to overall β-diversity, which remained relatively stable, genus-level analysis identified several differential signals. Notably, *Extibacter* showed the strongest exploratory non-response-associated pattern in this cohort. This taxon was undetectable in all baseline samples from the response group but present at non-zero abundance in 6 of 13 non-responder samples (Wilcoxon P = 0.0179). This distribution is consistent with an association between detection of *Extibacter* and non-response in this cohort, but it does not establish a causal or predictive role. Prior studies have shown that gut microbiota-mediated bile acid metabolism can influence hepatic antitumor immunity through the CXCL16–NKT cell axis (31). Furthermore, *Extibacter muris*, an anaerobic species studied in gnotobiotic mice, has been shown to produce secondary bile acids and influence liver physiology (32). These external findings provide a biological hypothesis only. The present study did not measure *Extibacter*-associated functions, bile acid profiles, metagenomic pathways, or host immune responses. Accordingly, *Extibacter* should be regarded as an exploratory genus-level signal requiring independent and functional validation.

Beyond *Extibacter*, the relative enrichment of the [*Ruminococ*c*us]_gnavus*_group in the non-response group is also notable. *Ruminococcus gnavus* is a commensal gut bacterium with complex biological traits that has garnered increasing attention in recent years. Distinct strains can exert divergent functional roles in mucosal utilization, carbohydrate metabolism, and inflammatory homeostasis (33). Prior work has demonstrated that certain strains are associated with mucin metabolism and inflammatory diseases, capable of producing pro-inflammatory polysaccharide antigens (34); other investigations further indicate that structural components such as capsular polysaccharides may profoundly modulate host immune responses (35). In the present study, the [*Ruminococcus]_gnavus*_group exhibited significant positive correlations with the “immune system diseases” and “carbohydrate metabolism” KEGG modules. Because these modules were inferred from 16S data and Ruminococcus gnavus is strain heterogeneous, the observed correlations should be regarded as exploratory and do not demonstrate mucosal disruption, metabolic dysregulation, or suppression of treatment efficacy.

Conversely, the *Eubacterium eligens*_group was enriched in the response group. Previous studies have reported that *Eubacterium eligens* can exhibit anti-inflammatory properties under pectin- and pectic-oligosaccharide-associated conditions, with links to IL-10-mediated immune responses (36). More broadly, gut microbiota-derived metabolites can influence antitumor immunity through multiple host pathways (37). However, the observed genus-level association does not demonstrate that this taxon exerted anti-inflammatory effects in these patients. Taken together, the differential taxa generate hypotheses about favorable and unfavorable microbial contexts, but the present data do not support a mechanistic model of microbiota-mediated response.

Because the overall microbial community showed little longitudinal change during the study period, baseline taxa were explored as candidate response-associated features. We evaluated an exploratory bivariate discriminatory model in which baseline *Extibacter* alone yielded an apparent AUC of 0.731, which increased to 0.777 when combined with AFP. Integrated microbial-clinical models have been investigated in HCC immunotherapy research (18–24). However, the present estimates were derived from the same small cohort, and bootstrap resampling assessed only internal stability. These findings do not establish validated predictive performance, clinical utility, or readiness for pretreatment stratification; external validation is required.

Several limitations should be acknowledged in the present study. First, this prospective multicenter exploratory cohort had a small sample size (n = 23). The reliability and generalizability of the differential genera and discriminatory analyses require validation in larger independent cohorts. Although bootstrap-based internal assessment was performed for the exploratory ROC analyses, the estimates were derived from the same small cohort and lacked external validation. Second, V3–V4 16S rRNA sequencing provides limited species- and strain-level resolution, constraining interpretation of strain-heterogeneous bacteria such as *Ruminococcus gnavus* (33–35). Functional interpretations involving bile acid metabolism and short-chain fatty acid production were based on literature and 16S-inferred profiles, without direct metagenomic, metabolomic, or experimental evidence. Third, residual confounding cannot be excluded. The parent trial partially restricted several major microbiota-relevant exposures including pretreatment systemic infection and systemic administration of corticosteroids and immunosuppressants, whereas microbiota-related factors including proton pump inhibitors, probiotics/prebiotics, lactulose, diet and recent gastrointestinal symptoms were not included in adjustment models.

Future studies should integrate shotgun metagenomic sequencing, targeted bile acid profiling, broader metabolomics, and mechanistic experiments to test the hypotheses generated here. Previous melanoma studies have shown that fecal microbiota transplantation can partially overcome anti-PD-1 resistance, illustrating the broader translational interest in microbiota-directed interventions (38, 39).

In this exploratory cohort of patients with HCC and PVTT receiving SBRT plus cadonilimab and lenvatinib, the overall gut microbiota structure showed limited separation between response groups and little longitudinal remodeling during treatment. Several genus-level taxa differed between responders and non-responders, and *Extibacter* was detected in a subset of non-responders but not in responders. These observations are associative and hypothesis-generating and require confirmation in larger independent cohorts. Because functional profiles were inferred from 16S rRNA data, this study does not establish microbial metabolic functions or causal mechanisms. Shotgun metagenomic sequencing, metabolomic profiling, and experimental validation will be needed to test the biological hypotheses generated by these findings.

## Data Availability

The original contributions presented in the study are included in the article/**Supplementary Material**. Further inquiries can be directed to the corresponding authors.
