# Supplementary material for: Association between gut microbiota characteristics and therapeutic efficacy of multimodal combination therapy in hepatocellular carcinoma with portal vein tumor thrombus: a secondary analysis of a prospective phase II trial
Source: Front Immunol. 2026 Aug 18;17:1864941. doi: 10.3389/fimmu.2026.1864941 (PMC13529949; doi:10.3389/fimmu.2026.1864941)
Supplement: Supplementary file 1 [file DataSheet1.docx]

Supplementary Material

# 1. Supplementary Figures

## 1.1. Figure S1

Figure S1 Quality-control workflow and read retention for 16S rRNA sequencing data

## 1.2. Figure S2

Figure S2 Rarefaction curves for alpha-diversity indices across samples

Note:A, observed_features; B, Shannon index; C, Faith's phylogenetic diversity (Faith's PD). All curves approached a plateau with increasing sequencing depth, indicating sufficient sequencing coverage for downstream diversity analyses.

## 1.3. Figure S3

Figure S3 Community composition at baseline in responders and non-responders

Note:A, stacked bar plot at the phylum level; B, stacked bar plot at the genus level.

## 1.4. Figure S4

Figure S4 Longitudinal changes in gut microbiota alpha diversity before and after combination therapy

Note:A, all patients; B, responders (group C); C, non-responders (group D).

## 1.5. Figure S5

Figure S5 Baseline between-group comparison of gut microbiota beta diversity

Note:A, weighted UniFrac PCoA; B, unweighted UniFrac PCoA Abbreviation: PCoA, principal coordinates analysis.

## 1.6. Figure S6

Figure S6 Correlation heatmap between differential genera and KEGG level 2 functional modules

Note:Heatmap colors represent Spearman's correlation coefficient rho (range, -1 to 1), with red indicating positive correlations and blue indicating negative correlations. Rows (KEGG level 2 pathways) and columns (differential genera) were ordered by hierarchical clustering. Significant associations after Benjamini-Hochberg correction are indicated in the corresponding annotations.

## 1.7. Figure S7

Figure S7 Overall paired comparison of gut microbiota beta diversity

Note:A, Bray-Curtis PCoA; B, weighted UniFrac PCoA; C, unweighted UniFrac PCoA. Abbreviation: PCoA, principal coordinates analysis.

## 1.8. Figure S8

Figure S8 Post-treatment between-group comparison of gut microbiota beta diversity

Note:A, Bray-Curtis PCoA; B, weighted UniFrac PCoA; C, unweighted UniFrac PCoA. Abbreviation: PCoA, principal coordinates analysis.

# 2. Supplementary Tables

## 2.1. Table S1

Table S1 Per-sample sequencing depth and DADA2 read-retention statistics

| Sample ID | Patient code | Time point | Input reads | Filtered reads | Passed filter (%) | Denoised reads | Merged reads | Merged (%) | Non-chimeric reads | Non-chimeric (%) | Rarefaction depth | Retained for diversity analysis |
| --- | --- | --- | --- | --- | --- | --- | --- | --- | --- | --- | --- | --- |
| A01 | 01 | Phase A (baseline) | 106551 | 98613 | 92.55% | 98158 | 45487 | 42.69% | 42187 | 39.59% | 5459 | Yes |
| A02 | 02 | Phase A (baseline) | 106377 | 97511 | 91.67% | 96860 | 16279 | 15.30% | 14179 | 13.33% | 5459 | Yes |
| A03 | 03 | Phase A (baseline) | 87541 | 81850 | 93.50% | 81398 | 52553 | 60.03% | 49078 | 56.06% | 5459 | Yes |
| A04 | 04 | Phase A (baseline) | 105994 | 98902 | 93.31% | 98343 | 31606 | 29.82% | 29627 | 27.95% | 5459 | Yes |
| A05 | 05 | Phase A (baseline) | 103096 | 94122 | 91.30% | 93688 | 5572 | 5.40% | 5459 | 5.30% | 5459 | Yes |
| A06 | 06 | Phase A (baseline) | 104310 | 97522 | 93.49% | 97064 | 37169 | 35.63% | 29230 | 28.02% | 5459 | Yes |
| A07 | 07 | Phase A (baseline) | 102015 | 95425 | 93.54% | 95081 | 20422 | 20.02% | 19816 | 19.42% | 5459 | Yes |
| A08 | 08 | Phase A (baseline) | 106076 | 98628 | 92.98% | 98149 | 38156 | 35.97% | 36563 | 34.47% | 5459 | Yes |
| A09 | 09 | Phase A (baseline) | 105996 | 98325 | 92.76% | 97768 | 49207 | 46.42% | 48600 | 45.85% | 5459 | Yes |
| A10 | 10 | Phase A (baseline) | 116936 | 109185 | 93.37% | 108864 | 50628 | 43.30% | 49661 | 42.47% | 5459 | Yes |
| A11 | 11 | Phase A (baseline) | 104101 | 95794 | 92.02% | 94968 | 27527 | 26.44% | 22969 | 22.06% | 5459 | Yes |
| A12 | 12 | Phase A (baseline) | 110246 | 101821 | 92.36% | 101313 | 10436 | 9.47% | 9651 | 8.75% | 5459 | Yes |
| A13 | 13 | Phase A (baseline) | 105739 | 98646 | 93.29% | 97766 | 48903 | 46.25% | 45989 | 43.49% | 5459 | Yes |
| A14 | 14 | Phase A (baseline) | 106474 | 99461 | 93.41% | 98772 | 27649 | 25.97% | 23907 | 22.45% | 5459 | Yes |
| A15 | 15 | Phase A (baseline) | 110011 | 103064 | 93.69% | 102777 | 41076 | 37.34% | 35939 | 32.67% | 5459 | Yes |
| A16 | 16 | Phase A (baseline) | 65894 | 61530 | 93.38% | 60816 | 10039 | 15.24% | 7921 | 12.02% | 5459 | Yes |
| A17 | 17 | Phase A (baseline) | 105956 | 96387 | 90.97% | 95895 | 18930 | 17.87% | 14008 | 13.22% | 5459 | Yes |
| A18 | 18 | Phase A (baseline) | 114374 | 105315 | 92.08% | 104261 | 49283 | 43.09% | 40463 | 35.38% | 5459 | Yes |
| A19 | 19 | Phase A (baseline) | 98800 | 90525 | 91.62% | 89673 | 31533 | 31.92% | 28167 | 28.51% | 5459 | Yes |
| A20 | 20 | Phase A (baseline) | 110768 | 100835 | 91.03% | 99625 | 64666 | 58.38% | 49741 | 44.91% | 5459 | Yes |
| A21 | 21 | Phase A (baseline) | 102816 | 93703 | 91.14% | 92791 | 28186 | 27.41% | 21085 | 20.51% | 5459 | Yes |
| A22 | 22 | Phase A (baseline) | 62256 | 57185 | 91.85% | 56657 | 25073 | 40.27% | 22691 | 36.45% | 5459 | Yes |
| A23 | 23 | Phase A (baseline) | 65274 | 60021 | 91.95% | 59723 | 7171 | 10.99% | 6373 | 9.76% | 5459 | Yes |
| B01 | 01 | Phase B (post-treatment) | 104958 | 97701 | 93.09% | 96902 | 42668 | 40.65% | 36567 | 34.84% | 5459 | Yes |
| B02 | 02 | Phase B (post-treatment) | 102470 | 95266 | 92.97% | 94748 | 36485 | 35.61% | 34134 | 33.31% | 5459 | Yes |
| B03 | 03 | Phase B (post-treatment) | 104495 | 96792 | 92.63% | 95969 | 45386 | 43.43% | 41867 | 40.07% | 5459 | Yes |
| B04 | 04 | Phase B (post-treatment) | 132076 | 120983 | 91.60% | 119922 | 26196 | 19.83% | 24150 | 18.28% | 5459 | Yes |
| B05 | 05 | Phase B (post-treatment) | 102941 | 95630 | 92.90% | 95132 | 15536 | 15.09% | 13687 | 13.30% | 5459 | Yes |
| B06 | 06 | Phase B (post-treatment) | 104137 | 96367 | 92.54% | 95511 | 34092 | 32.74% | 29783 | 28.60% | 5459 | Yes |
| B07 | 07 | Phase B (post-treatment) | 103833 | 96908 | 93.33% | 96492 | 39562 | 38.10% | 37689 | 36.30% | 5459 | Yes |
| B08 | 08 | Phase B (post-treatment) | 119488 | 111051 | 92.94% | 110657 | 32920 | 27.55% | 31435 | 26.31% | 5459 | Yes |
| B09 | 09 | Phase B (post-treatment) | 56644 | 52218 | 92.19% | 50333 | 17608 | 31.09% | 14799 | 26.13% | 5459 | Yes |
| B10 | 10 | Phase B (post-treatment) | 106072 | 98494 | 92.86% | 97423 | 39940 | 37.65% | 35056 | 33.05% | 5459 | Yes |
| B11 | 11 | Phase B (post-treatment) | 114670 | 107259 | 93.54% | 106595 | 30540 | 26.63% | 28935 | 25.23% | 5459 | Yes |
| B12 | 12 | Phase B (post-treatment) | 106280 | 98601 | 92.77% | 98210 | 50761 | 47.76% | 47125 | 44.34% | 5459 | Yes |
| B13 | 13 | Phase B (post-treatment) | 104605 | 97524 | 93.23% | 97143 | 20826 | 19.91% | 19639 | 18.77% | 5459 | Yes |
| B14 | 14 | Phase B (post-treatment) | 102379 | 95544 | 93.32% | 95147 | 29492 | 28.81% | 27702 | 27.06% | 5459 | Yes |
| B15 | 15 | Phase B (post-treatment) | 90937 | 84859 | 93.32% | 84682 | 64633 | 71.07% | 63955 | 70.33% | 5459 | Yes |
| B16 | 16 | Phase B (post-treatment) | 104358 | 97040 | 92.99% | 96620 | 67418 | 64.60% | 63062 | 60.43% | 5459 | Yes |
| B17 | 17 | Phase B (post-treatment) | 103353 | 93936 | 90.89% | 93206 | 20848 | 20.17% | 16542 | 16.01% | 5459 | Yes |
| B18 | 18 | Phase B (post-treatment) | 104309 | 96128 | 92.16% | 95194 | 68210 | 65.39% | 53550 | 51.34% | 5459 | Yes |
| B19 | 19 | Phase B (post-treatment) | 104445 | 96065 | 91.98% | 94794 | 48670 | 46.60% | 40614 | 38.89% | 5459 | Yes |
| B20 | 20 | Phase B (post-treatment) | 117851 | 108894 | 92.40% | 107920 | 60490 | 51.33% | 47060 | 39.93% | 5459 | Yes |
| B21 | 21 | Phase B (post-treatment) | 85593 | 78082 | 91.22% | 76842 | 25085 | 29.31% | 19528 | 22.81% | 5459 | Yes |
| B22 | 22 | Phase B (post-treatment) | 68409 | 62902 | 91.95% | 62293 | 23693 | 34.63% | 20888 | 30.53% | 5459 | Yes |
| B23 | 23 | Phase B (post-treatment) | 77068 | 70975 | 92.09% | 70474 | 10812 | 14.03% | 9132 | 11.85% | 5459 | Yes |

Note:Input reads represent demultiplexed paired-end reads before DADA2 processing. Filtered, denoised, merged, and non-chimeric reads were generated during the DADA2 workflow. The rarefaction depth was set at 5,459 reads per sample, corresponding to the lowest number of non-chimeric reads retained among all samples, allowing all 46 paired fecal samples to be included in downstream α- and β-diversity analyses.

## 2.2. Table S2

Table S2a Alpha-diversity results for baseline samples

|  | chao1 | faith_pd | observed_features | shannon_entropy | simpson |
| --- | --- | --- | --- | --- | --- |
| A16 | 136.000 | 9.574 | 136.000 | 4.704 | 0.911 |
| A13 | 260.556 | 13.675 | 259.000 | 4.721 | 0.862 |
| A15 | 91.000 | 7.228 | 91.000 | 3.720 | 0.871 |
| A11 | 192.724 | 11.602 | 192.000 | 4.902 | 0.916 |
| A12 | 98.000 | 7.719 | 98.000 | 4.644 | 0.932 |
| A02 | 155.769 | 9.506 | 155.000 | 5.350 | 0.959 |
| A08 | 186.000 | 15.129 | 186.000 | 5.064 | 0.937 |
| A06 | 88.100 | 5.884 | 86.000 | 3.423 | 0.860 |
| A05 | 199.100 | 13.232 | 199.000 | 5.605 | 0.925 |
| A04 | 131.000 | 8.235 | 131.000 | 3.129 | 0.748 |
| A07 | 78.000 | 6.650 | 78.000 | 3.871 | 0.893 |
| A03 | 171.000 | 12.871 | 171.000 | 4.788 | 0.927 |
| A09 | 304.200 | 17.643 | 304.000 | 5.644 | 0.941 |
| A14 | 153.000 | 8.971 | 153.000 | 4.192 | 0.838 |
| A01 | 147.000 | 9.410 | 147.000 | 2.738 | 0.592 |
| A10 | 99.000 | 8.416 | 99.000 | 3.718 | 0.858 |
| A17 | 113.063 | 7.982 | 113.000 | 3.611 | 0.823 |
| A18 | 154.400 | 8.296 | 153.000 | 4.688 | 0.924 |
| A19 | 179.077 | 11.511 | 178.000 | 3.983 | 0.856 |
| A20 | 195.400 | 10.265 | 194.000 | 5.018 | 0.951 |
| A21 | 154.526 | 8.908 | 154.000 | 4.568 | 0.900 |
| A22 | 134.000 | 7.689 | 134.000 | 3.666 | 0.746 |
| A23 | 73.429 | 5.487 | 73.000 | 3.237 | 0.744 |

Table S2b Alpha-diversity results for all paired samples

|  | chao1 | faith_pd | observed_features | shannon_entropy | simpson |
| --- | --- | --- | --- | --- | --- |
| A16 | 136.000 | 9.574 | 136.000 | 4.704 | 0.911 |
| B16 | 115.000 | 7.498 | 115.000 | 3.598 | 0.844 |
| A13 | 260.556 | 13.675 | 259.000 | 4.721 | 0.862 |
| B13 | 126.231 | 8.700 | 126.000 | 4.197 | 0.871 |
| A15 | 91.000 | 7.228 | 91.000 | 3.720 | 0.871 |
| B15 | 96.000 | 11.004 | 96.000 | 2.334 | 0.574 |
| A11 | 192.724 | 11.602 | 192.000 | 4.902 | 0.916 |
| B11 | 177.000 | 14.810 | 177.000 | 5.045 | 0.946 |
| A12 | 98.000 | 7.719 | 98.000 | 4.644 | 0.932 |
| B12 | 103.000 | 6.902 | 103.000 | 4.513 | 0.931 |
| A02 | 155.769 | 9.506 | 155.000 | 5.350 | 0.959 |
| B02 | 135.000 | 8.307 | 135.000 | 3.963 | 0.861 |
| A08 | 186.000 | 15.129 | 186.000 | 5.064 | 0.937 |
| B08 | 91.667 | 5.735 | 91.000 | 4.006 | 0.892 |
| A06 | 88.100 | 5.884 | 86.000 | 3.423 | 0.860 |
| B06 | 167.833 | 9.015 | 162.000 | 2.849 | 0.561 |
| A05 | 199.100 | 13.232 | 199.000 | 5.605 | 0.925 |
| B05 | 76.273 | 6.790 | 76.000 | 3.444 | 0.826 |
| A04 | 131.000 | 8.235 | 131.000 | 3.129 | 0.748 |
| B04 | 180.469 | 25.304 | 180.000 | 4.369 | 0.876 |
| A07 | 78.000 | 6.650 | 78.000 | 3.871 | 0.893 |
| B07 | 162.167 | 9.370 | 162.000 | 4.902 | 0.927 |
| A03 | 171.000 | 12.871 | 171.000 | 4.788 | 0.927 |
| B03 | 221.150 | 21.358 | 221.000 | 4.576 | 0.889 |
| A09 | 304.200 | 17.643 | 304.000 | 5.644 | 0.941 |
| B09 | 355.694 | 64.917 | 354.000 | 5.799 | 0.945 |
| A14 | 153.000 | 8.971 | 153.000 | 4.192 | 0.838 |
| B14 | 116.429 | 6.171 | 116.000 | 4.702 | 0.921 |
| A01 | 147.000 | 9.410 | 147.000 | 2.738 | 0.592 |
| B01 | 197.043 | 10.724 | 197.000 | 4.352 | 0.854 |
| A10 | 99.000 | 8.416 | 99.000 | 3.718 | 0.858 |
| B10 | 212.077 | 13.332 | 211.000 | 4.901 | 0.936 |
| B17 | 104.214 | 8.130 | 104.000 | 3.231 | 0.779 |
| A17 | 113.063 | 7.982 | 113.000 | 3.611 | 0.823 |
| A18 | 154.400 | 8.296 | 153.000 | 4.688 | 0.924 |
| B18 | 175.077 | 8.832 | 175.000 | 4.695 | 0.864 |
| A19 | 179.077 | 11.511 | 178.000 | 3.983 | 0.856 |
| B19 | 259.077 | 14.402 | 258.000 | 5.386 | 0.953 |
| A20 | 195.400 | 10.265 | 194.000 | 5.018 | 0.951 |
| B20 | 162.105 | 9.985 | 161.000 | 3.834 | 0.861 |
| A21 | 154.526 | 8.908 | 154.000 | 4.568 | 0.900 |
| B21 | 229.778 | 12.188 | 226.000 | 4.784 | 0.899 |
| B22 | 145.091 | 7.397 | 145.000 | 4.651 | 0.873 |
| A22 | 134.000 | 7.689 | 134.000 | 3.666 | 0.746 |
| B23 | 89.000 | 9.748 | 88.000 | 4.474 | 0.910 |
| A23 | 73.429 | 5.487 | 73.000 | 3.237 | 0.744 |

Table S2c Alpha-diversity results for responders (group C)

|  | chao1 | faith_pd | observed_features | shannon_entropy | simpson |
| --- | --- | --- | --- | --- | --- |
| A16 | 136.000 | 9.574 | 136.000 | 4.704 | 0.911 |
| B16 | 115.000 | 7.498 | 115.000 | 3.598 | 0.844 |
| A13 | 260.556 | 13.675 | 259.000 | 4.721 | 0.862 |
| B13 | 126.231 | 8.700 | 126.000 | 4.197 | 0.871 |
| A08 | 186.000 | 15.129 | 186.000 | 5.064 | 0.937 |
| B08 | 91.667 | 5.735 | 91.000 | 4.006 | 0.892 |
| A05 | 199.100 | 13.232 | 199.000 | 5.605 | 0.925 |
| B05 | 76.273 | 6.790 | 76.000 | 3.444 | 0.826 |
| A04 | 131.000 | 8.235 | 131.000 | 3.129 | 0.748 |
| B04 | 180.469 | 25.304 | 180.000 | 4.369 | 0.876 |
| A03 | 171.000 | 12.871 | 171.000 | 4.788 | 0.927 |
| B03 | 221.150 | 21.358 | 221.000 | 4.576 | 0.889 |
| A01 | 147.000 | 9.410 | 147.000 | 2.738 | 0.592 |
| B01 | 197.043 | 10.724 | 197.000 | 4.352 | 0.854 |
| B17 | 104.214 | 8.130 | 104.000 | 3.231 | 0.779 |
| A17 | 113.063 | 7.982 | 113.000 | 3.611 | 0.823 |
| A19 | 179.077 | 11.511 | 178.000 | 3.983 | 0.856 |
| B19 | 259.077 | 14.402 | 258.000 | 5.386 | 0.953 |
| A20 | 195.400 | 10.265 | 194.000 | 5.018 | 0.951 |
| B20 | 162.105 | 9.985 | 161.000 | 3.834 | 0.861 |

Table S2d Alpha-diversity results for non-responders (group D)

|  | chao1 | faith_pd | observed_features | shannon_entropy | simpson |
| --- | --- | --- | --- | --- | --- |
| A15 | 91.000 | 7.228 | 91.000 | 3.720 | 0.871 |
| B15 | 96.000 | 11.004 | 96.000 | 2.334 | 0.574 |
| A11 | 192.724 | 11.602 | 192.000 | 4.902 | 0.916 |
| B11 | 177.000 | 14.810 | 177.000 | 5.045 | 0.946 |
| A12 | 98.000 | 7.719 | 98.000 | 4.644 | 0.932 |
| B12 | 103.000 | 6.902 | 103.000 | 4.513 | 0.931 |
| A02 | 155.769 | 9.506 | 155.000 | 5.350 | 0.959 |
| B02 | 135.000 | 8.307 | 135.000 | 3.963 | 0.861 |
| A06 | 88.100 | 5.884 | 86.000 | 3.423 | 0.860 |
| B06 | 167.833 | 9.015 | 162.000 | 2.849 | 0.561 |
| A07 | 78.000 | 6.650 | 78.000 | 3.871 | 0.893 |
| B07 | 162.167 | 9.370 | 162.000 | 4.902 | 0.927 |
| A09 | 304.200 | 17.643 | 304.000 | 5.644 | 0.941 |
| B09 | 355.694 | 64.917 | 354.000 | 5.799 | 0.945 |
| A14 | 153.000 | 8.971 | 153.000 | 4.192 | 0.838 |
| B14 | 116.429 | 6.171 | 116.000 | 4.702 | 0.921 |
| A10 | 99.000 | 8.416 | 99.000 | 3.718 | 0.858 |
| B10 | 212.077 | 13.332 | 211.000 | 4.901 | 0.936 |
| A18 | 154.400 | 8.296 | 153.000 | 4.688 | 0.924 |
| B18 | 175.077 | 8.832 | 175.000 | 4.695 | 0.864 |
| A21 | 154.526 | 8.908 | 154.000 | 4.568 | 0.900 |
| B21 | 229.778 | 12.188 | 226.000 | 4.784 | 0.899 |
| B22 | 145.091 | 7.397 | 145.000 | 4.651 | 0.873 |
| A22 | 134.000 | 7.689 | 134.000 | 3.666 | 0.746 |
| B23 | 89.000 | 9.748 | 88.000 | 4.474 | 0.910 |
| A23 | 73.429 | 5.487 | 73.000 | 3.237 | 0.744 |

## 2.3. Table S3

Table S3 PERMANOVA results for baseline gut microbiota beta diversity between response group (Pre_C) and non-response group (Pre_D)

| Distance metric | Group 1 | Group 2 | Sample size | Permutations | pseudo-F | P  value | Q  value |
| --- | --- | --- | --- | --- | --- | --- | --- |
| Bray-Curtis | Pre_C | Pre_D | 23 | 999 | 0.918495 | 0.586 | 0.586 |
| weighted UniFrac | Pre_C | Pre_D | 23 | 999 | 0.855415 | 0.572 | 0.572 |
| unweighted UniFrac | Pre_C | Pre_D | 23 | 999 | 0.968942 | 0.514 | 0.514 |

## 2.4. Table S4

Table S4a Relative abundances of KEGG level 2 functional modules at baseline

|  | A01 | A02 | A03 | A04 | A05 | A06 | A07 | A08 | A09 | A10 | A11 | A12 | A13 | A14 | A15 | A16 | A17 | A18 | A19 | A20 | A21 | A22 | A23 |
| --- | --- | --- | --- | --- | --- | --- | --- | --- | --- | --- | --- | --- | --- | --- | --- | --- | --- | --- | --- | --- | --- | --- | --- |
| Cell growth and death | 1.869E-02 | 2.074E-02 | 2.008E-02 | 1.840E-02 | 1.985E-02 | 1.964E-02 | 1.851E-02 | 1.831E-02 | 1.959E-02 | 1.924E-02 | 1.918E-02 | 1.901E-02 | 2.019E-02 | 1.858E-02 | 1.848E-02 | 2.008E-02 | 2.014E-02 | 1.993E-02 | 1.888E-02 | 1.991E-02 | 1.941E-02 | 1.661E-02 | 2.059E-02 |
| Cell motility | 6.444E-02 | 1.675E-02 | 2.133E-02 | 2.821E-02 | 2.908E-02 | 1.128E-02 | 3.603E-02 | 2.523E-02 | 3.821E-02 | 8.714E-03 | 2.508E-02 | 3.171E-02 | 2.090E-02 | 4.706E-02 | 1.484E-02 | 3.451E-02 | 1.255E-02 | 1.402E-02 | 1.092E-02 | 2.486E-02 | 4.225E-02 | 1.664E-02 | 1.067E-02 |
| Cellular community - eukaryotes | 1.576E-07 | 4.061E-07 | 0.000E+00 | 6.293E-08 | 1.077E-05 | 0.000E+00 | 0.000E+00 | 0.000E+00 | 6.540E-07 | 3.767E-08 | 3.720E-08 | 0.000E+00 | 1.731E-07 | 2.620E-07 | 0.000E+00 | 0.000E+00 | 4.906E-07 | 0.000E+00 | 0.000E+00 | 0.000E+00 | 0.000E+00 | 8.444E-08 | 0.000E+00 |
| Cellular community - prokaryotes | 1.590E-02 | 1.402E-02 | 1.399E-02 | 1.416E-02 | 1.339E-02 | 1.455E-02 | 1.529E-02 | 1.302E-02 | 1.486E-02 | 1.271E-02 | 1.441E-02 | 1.422E-02 | 1.384E-02 | 1.549E-02 | 1.380E-02 | 1.442E-02 | 1.385E-02 | 1.300E-02 | 1.287E-02 | 1.417E-02 | 1.493E-02 | 1.126E-02 | 1.343E-02 |
| Transport and catabolism | 1.428E-03 | 1.988E-03 | 1.977E-03 | 1.579E-03 | 2.558E-03 | 2.383E-03 | 2.019E-03 | 2.564E-03 | 1.856E-03 | 1.694E-03 | 1.605E-03 | 2.000E-03 | 1.948E-03 | 1.701E-03 | 2.281E-03 | 2.066E-03 | 2.327E-03 | 1.934E-03 | 1.313E-03 | 1.981E-03 | 1.865E-03 | 4.172E-03 | 2.386E-03 |
| Membrane transport | 1.574E-02 | 2.087E-02 | 1.769E-02 | 1.892E-02 | 1.932E-02 | 2.542E-02 | 2.261E-02 | 1.684E-02 | 1.670E-02 | 2.232E-02 | 2.062E-02 | 1.872E-02 | 1.862E-02 | 1.936E-02 | 2.077E-02 | 1.673E-02 | 2.103E-02 | 2.147E-02 | 2.385E-02 | 1.803E-02 | 1.721E-02 | 1.440E-02 | 1.850E-02 |
| Signal transduction | 8.169E-03 | 8.354E-03 | 7.968E-03 | 9.494E-03 | 9.911E-03 | 8.535E-03 | 9.591E-03 | 8.823E-03 | 8.432E-03 | 8.197E-03 | 8.338E-03 | 8.425E-03 | 8.131E-03 | 8.450E-03 | 8.844E-03 | 8.130E-03 | 8.305E-03 | 7.988E-03 | 9.055E-03 | 8.175E-03 | 8.201E-03 | 9.348E-03 | 7.953E-03 |
| Signaling molecules and interaction | 6.245E-07 | 1.609E-06 | 0.000E+00 | 2.493E-07 | 3.733E-06 | 0.000E+00 | 0.000E+00 | 0.000E+00 | 2.524E-07 | 1.492E-07 | 0.000E+00 | 0.000E+00 | 6.083E-07 | 1.038E-06 | 0.000E+00 | 0.000E+00 | 1.944E-06 | 0.000E+00 | 0.000E+00 | 0.000E+00 | 0.000E+00 | 3.345E-07 | 0.000E+00 |
| Folding, sorting and degradation | 3.365E-02 | 3.299E-02 | 3.500E-02 | 3.305E-02 | 3.564E-02 | 3.365E-02 | 3.223E-02 | 3.262E-02 | 3.470E-02 | 3.350E-02 | 3.331E-02 | 3.159E-02 | 3.472E-02 | 3.264E-02 | 3.079E-02 | 3.540E-02 | 3.297E-02 | 3.393E-02 | 3.359E-02 | 3.245E-02 | 3.397E-02 | 2.903E-02 | 3.567E-02 |
| Replication and repair | 5.880E-02 | 5.987E-02 | 5.825E-02 | 5.986E-02 | 6.153E-02 | 5.867E-02 | 5.637E-02 | 5.870E-02 | 5.914E-02 | 5.669E-02 | 5.859E-02 | 5.798E-02 | 6.114E-02 | 5.675E-02 | 5.360E-02 | 6.056E-02 | 5.821E-02 | 5.833E-02 | 6.014E-02 | 5.859E-02 | 5.917E-02 | 5.222E-02 | 5.960E-02 |
| Transcription | 3.444E-03 | 3.363E-03 | 3.425E-03 | 3.102E-03 | 3.859E-03 | 3.404E-03 | 3.245E-03 | 3.422E-03 | 3.430E-03 | 3.478E-03 | 3.613E-03 | 3.450E-03 | 3.617E-03 | 3.374E-03 | 2.935E-03 | 3.574E-03 | 3.190E-03 | 3.457E-03 | 3.455E-03 | 3.283E-03 | 3.415E-03 | 3.177E-03 | 3.488E-03 |
| Translation | 3.564E-02 | 3.340E-02 | 3.452E-02 | 3.646E-02 | 3.609E-02 | 3.416E-02 | 3.277E-02 | 3.515E-02 | 3.501E-02 | 3.691E-02 | 3.486E-02 | 3.304E-02 | 3.546E-02 | 3.394E-02 | 2.859E-02 | 3.532E-02 | 3.294E-02 | 3.601E-02 | 3.899E-02 | 3.440E-02 | 3.484E-02 | 3.201E-02 | 3.445E-02 |
| Cancer: overview | 5.212E-03 | 5.416E-03 | 5.181E-03 | 5.943E-03 | 6.597E-03 | 5.356E-03 | 5.585E-03 | 5.599E-03 | 5.255E-03 | 5.105E-03 | 5.139E-03 | 5.339E-03 | 5.296E-03 | 5.103E-03 | 5.149E-03 | 5.546E-03 | 5.272E-03 | 5.380E-03 | 5.385E-03 | 5.279E-03 | 5.255E-03 | 6.046E-03 | 5.460E-03 |
| Cancer: specific types | 5.846E-04 | 5.447E-04 | 5.562E-04 | 5.878E-04 | 8.136E-04 | 5.710E-04 | 5.510E-04 | 4.942E-04 | 5.692E-04 | 5.633E-04 | 6.095E-04 | 5.953E-04 | 6.361E-04 | 5.657E-04 | 5.348E-04 | 6.685E-04 | 5.555E-04 | 5.381E-04 | 6.296E-04 | 5.199E-04 | 5.638E-04 | 5.287E-04 | 5.778E-04 |
| Cardiovascular disease | 1.836E-03 | 1.579E-03 | 1.887E-03 | 1.858E-03 | 1.646E-03 | 1.776E-03 | 1.624E-03 | 1.661E-03 | 1.810E-03 | 1.876E-03 | 1.758E-03 | 1.696E-03 | 1.787E-03 | 1.758E-03 | 1.701E-03 | 1.856E-03 | 1.748E-03 | 1.757E-03 | 1.779E-03 | 1.797E-03 | 1.842E-03 | 1.406E-03 | 1.949E-03 |
| Drug resistance: antimicrobial | 2.173E-02 | 2.185E-02 | 2.334E-02 | 2.615E-02 | 2.062E-02 | 2.434E-02 | 2.327E-02 | 2.316E-02 | 2.266E-02 | 2.366E-02 | 2.261E-02 | 2.285E-02 | 2.223E-02 | 2.159E-02 | 2.527E-02 | 2.168E-02 | 2.150E-02 | 2.304E-02 | 2.454E-02 | 2.207E-02 | 2.202E-02 | 2.294E-02 | 2.339E-02 |
| Drug resistance: antineoplastic | 7.198E-03 | 7.716E-03 | 7.872E-03 | 7.133E-03 | 7.739E-03 | 8.470E-03 | 7.960E-03 | 7.634E-03 | 7.865E-03 | 7.632E-03 | 8.302E-03 | 7.779E-03 | 8.396E-03 | 7.357E-03 | 6.582E-03 | 7.942E-03 | 7.588E-03 | 8.075E-03 | 7.805E-03 | 7.533E-03 | 7.370E-03 | 7.779E-03 | 8.111E-03 |
| Endocrine and metabolic disease | 4.871E-03 | 4.587E-03 | 4.785E-03 | 5.376E-03 | 5.839E-03 | 4.729E-03 | 4.798E-03 | 5.094E-03 | 4.907E-03 | 5.013E-03 | 4.562E-03 | 4.606E-03 | 4.988E-03 | 4.578E-03 | 4.127E-03 | 5.039E-03 | 4.588E-03 | 5.019E-03 | 5.353E-03 | 4.844E-03 | 4.851E-03 | 4.894E-03 | 4.924E-03 |
| Immune disease | 1.084E-03 | 1.476E-03 | 1.168E-03 | 1.042E-03 | 1.385E-03 | 1.607E-03 | 1.873E-03 | 1.108E-03 | 1.278E-03 | 5.478E-04 | 1.340E-03 | 1.127E-03 | 7.127E-04 | 1.319E-03 | 1.532E-03 | 7.541E-04 | 1.478E-03 | 8.832E-04 | 3.248E-04 | 1.178E-03 | 1.321E-03 | 5.635E-04 | 7.747E-04 |
| Infectious disease: bacterial | 6.853E-03 | 4.275E-03 | 4.593E-03 | 4.970E-03 | 5.656E-03 | 3.972E-03 | 4.789E-03 | 5.439E-03 | 5.130E-03 | 4.228E-03 | 5.028E-03 | 4.947E-03 | 4.347E-03 | 5.562E-03 | 4.374E-03 | 5.234E-03 | 3.752E-03 | 4.305E-03 | 4.226E-03 | 4.718E-03 | 5.738E-03 | 3.928E-03 | 4.336E-03 |
| Infectious disease: parasitic | 3.074E-05 | 8.964E-05 | 8.855E-05 | 3.500E-05 | 5.834E-04 | 2.267E-05 | 4.574E-05 | 5.252E-05 | 4.546E-05 | 2.168E-04 | 1.041E-04 | 1.121E-04 | 8.326E-05 | 3.902E-05 | 1.968E-04 | 6.111E-05 | 1.415E-04 | 1.708E-04 | 4.345E-05 | 1.970E-04 | 5.735E-05 | 1.826E-05 | 1.162E-04 |
| Infectious disease: viral | 1.889E-04 | 1.787E-04 | 1.846E-04 | 1.934E-04 | 3.453E-04 | 1.856E-04 | 2.314E-04 | 1.891E-04 | 1.954E-04 | 1.965E-04 | 1.854E-04 | 1.748E-04 | 1.931E-04 | 1.799E-04 | 1.560E-04 | 2.016E-04 | 1.800E-04 | 2.110E-04 | 2.065E-04 | 1.982E-04 | 1.930E-04 | 1.710E-04 | 1.895E-04 |
| Neurodegenerative disease | 4.233E-04 | 1.049E-03 | 5.746E-04 | 4.030E-04 | 1.201E-03 | 2.273E-03 | 1.261E-03 | 6.560E-04 | 4.055E-04 | 5.447E-04 | 7.981E-04 | 8.510E-04 | 5.081E-04 | 7.020E-04 | 1.417E-03 | 5.477E-04 | 1.031E-03 | 5.257E-04 | 5.511E-04 | 6.787E-04 | 8.743E-04 | 1.188E-03 | 5.476E-04 |
| Substance dependence | 3.846E-07 | 1.038E-06 | 0.000E+00 | 0.000E+00 | 1.304E-04 | 0.000E+00 | 4.178E-04 | 7.478E-06 | 2.553E-05 | 5.617E-07 | 0.000E+00 | 1.032E-06 | 1.792E-07 | 1.278E-06 | 1.720E-07 | 0.000E+00 | 3.511E-07 | 0.000E+00 | 0.000E+00 | 5.188E-07 | 6.251E-07 | 0.000E+00 | 0.000E+00 |
| Amino acid metabolism | 1.167E-01 | 1.155E-01 | 1.162E-01 | 1.145E-01 | 1.065E-01 | 1.140E-01 | 1.087E-01 | 1.086E-01 | 1.152E-01 | 1.230E-01 | 1.114E-01 | 1.119E-01 | 1.160E-01 | 1.136E-01 | 1.124E-01 | 1.168E-01 | 1.176E-01 | 1.200E-01 | 1.214E-01 | 1.198E-01 | 1.154E-01 | 1.099E-01 | 1.183E-01 |
| Biosynthesis of other secondary metabolites | 6.194E-02 | 6.694E-02 | 6.815E-02 | 6.639E-02 | 5.230E-02 | 6.482E-02 | 6.302E-02 | 6.285E-02 | 6.522E-02 | 6.848E-02 | 6.521E-02 | 6.438E-02 | 6.281E-02 | 6.457E-02 | 7.227E-02 | 6.295E-02 | 6.652E-02 | 6.574E-02 | 6.424E-02 | 6.581E-02 | 6.262E-02 | 6.258E-02 | 6.633E-02 |
| Carbohydrate metabolism | 1.034E-01 | 1.210E-01 | 1.085E-01 | 1.071E-01 | 1.065E-01 | 1.233E-01 | 1.186E-01 | 1.021E-01 | 1.042E-01 | 1.158E-01 | 1.133E-01 | 1.120E-01 | 1.102E-01 | 1.132E-01 | 1.241E-01 | 1.030E-01 | 1.198E-01 | 1.154E-01 | 1.145E-01 | 1.110E-01 | 1.073E-01 | 1.012E-01 | 1.074E-01 |
| Chemical structure transformation maps | 1.272E-05 | 2.862E-05 | 5.271E-05 | 4.812E-05 | 4.430E-04 | 0.000E+00 | 5.669E-05 | 7.098E-03 | 1.343E-04 | 3.847E-04 | 1.409E-04 | 8.537E-06 | 1.976E-05 | 5.640E-05 | 4.855E-04 | 4.585E-05 | 1.917E-04 | 1.126E-06 | 6.573E-05 | 5.236E-05 | 6.205E-06 | 2.003E-02 | 2.798E-05 |
| Energy metabolism | 4.487E-02 | 4.710E-02 | 4.927E-02 | 4.581E-02 | 4.579E-02 | 4.654E-02 | 4.600E-02 | 4.609E-02 | 4.739E-02 | 4.907E-02 | 4.694E-02 | 4.649E-02 | 4.673E-02 | 4.574E-02 | 4.766E-02 | 4.634E-02 | 4.434E-02 | 4.959E-02 | 4.764E-02 | 4.600E-02 | 4.476E-02 | 4.056E-02 | 4.795E-02 |
| Global and overview maps | 6.473E-02 | 6.257E-02 | 6.518E-02 | 6.290E-02 | 5.789E-02 | 6.205E-02 | 6.073E-02 | 6.064E-02 | 6.436E-02 | 6.698E-02 | 6.166E-02 | 6.138E-02 | 6.486E-02 | 6.303E-02 | 6.123E-02 | 6.547E-02 | 6.347E-02 | 6.647E-02 | 6.559E-02 | 6.461E-02 | 6.363E-02 | 5.777E-02 | 6.673E-02 |
| Glycan biosynthesis and metabolism | 3.202E-02 | 3.796E-02 | 3.456E-02 | 3.332E-02 | 3.439E-02 | 3.811E-02 | 3.551E-02 | 5.210E-02 | 3.353E-02 | 3.137E-02 | 3.414E-02 | 3.433E-02 | 3.346E-02 | 3.523E-02 | 4.456E-02 | 3.375E-02 | 4.186E-02 | 3.137E-02 | 2.802E-02 | 3.420E-02 | 3.550E-02 | 9.010E-02 | 3.975E-02 |
| Lipid metabolism | 4.096E-02 | 4.192E-02 | 4.494E-02 | 4.446E-02 | 4.226E-02 | 4.288E-02 | 4.834E-02 | 4.162E-02 | 4.425E-02 | 4.470E-02 | 4.282E-02 | 4.812E-02 | 4.545E-02 | 4.176E-02 | 4.595E-02 | 4.383E-02 | 4.673E-02 | 4.502E-02 | 4.480E-02 | 4.431E-02 | 4.403E-02 | 3.784E-02 | 4.559E-02 |
| Metabolism of cofactors and vitamins | 8.798E-02 | 8.910E-02 | 9.625E-02 | 8.263E-02 | 9.829E-02 | 8.615E-02 | 8.637E-02 | 9.785E-02 | 9.367E-02 | 8.930E-02 | 9.334E-02 | 9.186E-02 | 9.616E-02 | 8.939E-02 | 8.504E-02 | 9.665E-02 | 9.166E-02 | 9.172E-02 | 8.375E-02 | 9.343E-02 | 9.148E-02 | 1.043E-01 | 9.906E-02 |
| Metabolism of other amino acids | 6.505E-02 | 7.087E-02 | 6.857E-02 | 7.182E-02 | 7.727E-02 | 7.240E-02 | 6.577E-02 | 7.178E-02 | 6.651E-02 | 7.056E-02 | 7.481E-02 | 7.306E-02 | 7.082E-02 | 6.717E-02 | 7.100E-02 | 6.774E-02 | 6.910E-02 | 6.924E-02 | 7.394E-02 | 7.050E-02 | 6.903E-02 | 5.950E-02 | 6.829E-02 |
| Metabolism of terpenoids and polyketides | 2.425E-02 | 2.643E-02 | 2.538E-02 | 2.777E-02 | 2.733E-02 | 2.925E-02 | 2.764E-02 | 2.473E-02 | 2.462E-02 | 2.746E-02 | 2.510E-02 | 2.503E-02 | 2.386E-02 | 2.453E-02 | 2.702E-02 | 2.442E-02 | 2.724E-02 | 2.651E-02 | 2.836E-02 | 2.568E-02 | 2.553E-02 | 2.319E-02 | 2.390E-02 |
| Nucleotide metabolism | 1.638E-02 | 1.701E-02 | 1.705E-02 | 1.824E-02 | 1.749E-02 | 1.757E-02 | 1.694E-02 | 1.688E-02 | 1.711E-02 | 1.727E-02 | 1.720E-02 | 1.709E-02 | 1.739E-02 | 1.658E-02 | 1.638E-02 | 1.730E-02 | 1.669E-02 | 1.730E-02 | 1.820E-02 | 1.719E-02 | 1.682E-02 | 1.466E-02 | 1.739E-02 |
| Xenobiotics biodegradation and metabolism | 1.436E-02 | 2.285E-02 | 2.086E-02 | 2.395E-02 | 2.267E-02 | 1.926E-02 | 2.115E-02 | 2.024E-02 | 2.013E-02 | 2.129E-02 | 2.255E-02 | 2.320E-02 | 2.231E-02 | 1.835E-02 | 2.705E-02 | 1.928E-02 | 2.226E-02 | 2.063E-02 | 2.201E-02 | 2.189E-02 | 1.728E-02 | 1.723E-02 | 2.141E-02 |
| Aging | 3.394E-03 | 3.625E-03 | 3.516E-03 | 3.706E-03 | 5.899E-03 | 3.949E-03 | 4.475E-03 | 4.168E-03 | 3.646E-03 | 3.755E-03 | 3.681E-03 | 3.584E-03 | 3.528E-03 | 3.421E-03 | 3.585E-03 | 3.696E-03 | 3.453E-03 | 3.887E-03 | 4.092E-03 | 3.617E-03 | 3.621E-03 | 5.323E-03 | 3.515E-03 |
| Circulatory system | 1.060E-07 | 7.156E-07 | 0.000E+00 | 3.105E-07 | 1.353E-05 | 1.485E-07 | 4.049E-07 | 1.225E-07 | 5.981E-07 | 8.828E-07 | 0.000E+00 | 5.122E-06 | 1.482E-07 | 0.000E+00 | 0.000E+00 | 0.000E+00 | 0.000E+00 | 0.000E+00 | 0.000E+00 | 4.721E-07 | 7.239E-07 | 0.000E+00 | 1.632E-06 |
| Development and regeneration | 0.000E+00 | 0.000E+00 | 0.000E+00 | 0.000E+00 | 6.076E-07 | 0.000E+00 | 0.000E+00 | 0.000E+00 | 0.000E+00 | 0.000E+00 | 0.000E+00 | 0.000E+00 | 0.000E+00 | 0.000E+00 | 0.000E+00 | 0.000E+00 | 0.000E+00 | 0.000E+00 | 0.000E+00 | 0.000E+00 | 0.000E+00 | 0.000E+00 | 0.000E+00 |
| Digestive system | 8.278E-05 | 5.330E-05 | 2.533E-05 | 3.758E-05 | 9.517E-04 | 6.854E-06 | 7.094E-05 | 3.366E-05 | 6.382E-05 | 4.246E-04 | 5.621E-04 | 2.545E-05 | 7.661E-06 | 7.514E-05 | 6.345E-05 | 1.791E-04 | 3.019E-04 | 2.774E-04 | 4.871E-05 | 1.200E-04 | 4.551E-05 | 3.292E-05 | 5.979E-05 |
| Endocrine system | 8.044E-03 | 7.967E-03 | 8.446E-03 | 9.567E-03 | 9.632E-03 | 7.667E-03 | 7.764E-03 | 8.325E-03 | 8.588E-03 | 8.786E-03 | 8.166E-03 | 8.412E-03 | 9.330E-03 | 7.958E-03 | 7.159E-03 | 8.929E-03 | 7.962E-03 | 8.775E-03 | 9.515E-03 | 8.548E-03 | 8.384E-03 | 7.893E-03 | 8.848E-03 |
| Environmental adaptation | 2.829E-03 | 1.382E-03 | 1.754E-03 | 1.860E-03 | 1.592E-03 | 1.354E-03 | 1.876E-03 | 1.673E-03 | 2.085E-03 | 1.413E-03 | 1.731E-03 | 1.824E-03 | 1.728E-03 | 2.346E-03 | 1.301E-03 | 2.118E-03 | 1.328E-03 | 1.540E-03 | 1.480E-03 | 1.641E-03 | 2.243E-03 | 1.096E-03 | 1.541E-03 |
| Excretory system | 2.624E-04 | 6.150E-04 | 3.446E-04 | 2.034E-03 | 3.425E-03 | 1.343E-04 | 3.523E-04 | 1.587E-03 | 5.871E-04 | 5.988E-04 | 1.110E-03 | 1.139E-03 | 1.103E-03 | 6.545E-04 | 8.089E-04 | 4.769E-04 | 3.521E-04 | 3.322E-04 | 1.823E-03 | 5.396E-04 | 4.432E-04 | 2.950E-03 | 1.659E-04 |
| Immune system | 4.096E-03 | 2.920E-03 | 3.380E-03 | 3.540E-03 | 2.552E-03 | 3.084E-03 | 3.245E-03 | 3.052E-03 | 3.545E-03 | 3.219E-03 | 3.307E-03 | 3.239E-03 | 3.320E-03 | 3.590E-03 | 3.006E-03 | 3.544E-03 | 2.887E-03 | 3.064E-03 | 3.364E-03 | 3.199E-03 | 3.687E-03 | 2.810E-03 | 3.173E-03 |
| Nervous system | 2.732E-03 | 3.002E-03 | 3.130E-03 | 3.337E-03 | 3.042E-03 | 2.528E-03 | 2.367E-03 | 2.827E-03 | 3.086E-03 | 3.122E-03 | 2.750E-03 | 2.730E-03 | 3.136E-03 | 2.667E-03 | 2.924E-03 | 3.113E-03 | 2.951E-03 | 3.146E-03 | 3.227E-03 | 2.966E-03 | 2.867E-03 | 2.812E-03 | 3.430E-03 |

Table S4b Dunn's test results for KEGG level 2 functional modules at baseline

| Feature | KW_pvalue | DunnTest_comparison | DunnTest_Z | DunnTest_PValue | DunnTest_PValueBonferroniAdjusted |
| --- | --- | --- | --- | --- | --- |
| Cell growth and death | 0.577 | Pre_C - Pre_D | 0.558 | 0.288 | 0.288 |
| Cell motility | 0.664 | Pre_C - Pre_D | 0.434 | 0.332 | 0.332 |
| Cellular community - eukaryotes | 0.664 | Pre_C - Pre_D | 0.435 | 0.332 | 0.332 |
| Cellular community - prokaryotes | 0.577 | Pre_C - Pre_D | -0.558 | 0.288 | 0.288 |
| Transport and catabolism | 0.804 | Pre_C - Pre_D | -0.248 | 0.402 | 0.402 |
| Membrane transport | 0.239 | Pre_C - Pre_D | -1.178 | 0.119 | 0.119 |
| Signal transduction | 0.710 | Pre_C - Pre_D | -0.372 | 0.355 | 0.355 |
| Signaling molecules and interaction | 0.411 | Pre_C - Pre_D | 0.822 | 0.206 | 0.206 |
| Folding, sorting and degradation | 0.264 | Pre_C - Pre_D | 1.116 | 0.132 | 0.132 |
| Replication and repair | 0.022 | Pre_C - Pre_D | 2.295 | 0.011 | 0.011 |
| Transcription | 0.535 | Pre_C - Pre_D | 0.620 | 0.268 | 0.268 |
| Translation | 0.035 | Pre_C - Pre_D | 2.109 | 0.017 | 0.017 |
| Cancer: overview | 0.264 | Pre_C - Pre_D | 1.116 | 0.132 | 0.132 |
| Cancer: specific types | 0.239 | Pre_C - Pre_D | 1.178 | 0.119 | 0.119 |
| Cardiovascular disease | 0.292 | Pre_C - Pre_D | 1.054 | 0.146 | 0.146 |
| Drug resistance: antimicrobial | 0.352 | Pre_C - Pre_D | -0.930 | 0.176 | 0.176 |
| Drug resistance: antineoplastic | 0.420 | Pre_C - Pre_D | -0.806 | 0.210 | 0.210 |
| Endocrine and metabolic disease | 0.026 | Pre_C - Pre_D | 2.233 | 0.013 | 0.013 |
| Immune disease | 0.215 | Pre_C - Pre_D | -1.240 | 0.107 | 0.107 |
| Infectious disease: bacterial | 0.420 | Pre_C - Pre_D | 0.806 | 0.210 | 0.210 |
| Infectious disease: parasitic | 1.000 | Pre_C - Pre_D | 0.000 | 0.500 | 0.500 |
| Infectious disease: viral | 0.154 | Pre_C - Pre_D | 1.426 | 0.077 | 0.077 |
| Neurodegenerative disease | 0.121 | Pre_C - Pre_D | -1.550 | 0.061 | 0.061 |
| Substance dependence | 0.609 | Pre_C - Pre_D | -0.512 | 0.304 | 0.304 |
| Amino acid metabolism | 0.352 | Pre_C - Pre_D | 0.930 | 0.176 | 0.176 |
| Biosynthesis of other secondary metabolites | 0.321 | Pre_C - Pre_D | -0.992 | 0.161 | 0.161 |
| Carbohydrate metabolism | 0.072 | Pre_C - Pre_D | -1.799 | 0.036 | 0.036 |
| Chemical structure transformation maps | 0.620 | Pre_C - Pre_D | 0.496 | 0.310 | 0.310 |
| Energy metabolism | 0.292 | Pre_C - Pre_D | -1.054 | 0.146 | 0.146 |
| Global and overview maps | 0.457 | Pre_C - Pre_D | 0.744 | 0.228 | 0.228 |
| Glycan biosynthesis and metabolism | 0.239 | Pre_C - Pre_D | -1.178 | 0.119 | 0.119 |
| Lipid metabolism | 0.756 | Pre_C - Pre_D | -0.310 | 0.378 | 0.378 |
| Metabolism of cofactors and vitamins | 0.535 | Pre_C - Pre_D | 0.620 | 0.268 | 0.268 |
| Metabolism of other amino acids | 0.495 | Pre_C - Pre_D | 0.682 | 0.248 | 0.248 |
| Metabolism of terpenoids and polyketides | 0.951 | Pre_C - Pre_D | 0.062 | 0.475 | 0.475 |
| Nucleotide metabolism | 0.264 | Pre_C - Pre_D | 1.116 | 0.132 | 0.132 |
| Xenobiotics biodegradation and metabolism | 0.620 | Pre_C - Pre_D | 0.496 | 0.310 | 0.310 |
| Aging | 0.756 | Pre_C - Pre_D | -0.310 | 0.378 | 0.378 |
| Circulatory system | 0.371 | Pre_C - Pre_D | -0.895 | 0.185 | 0.185 |
| Development and regeneration | 0.254 | Pre_C - Pre_D | 1.140 | 0.127 | 0.127 |
| Digestive system | 0.951 | Pre_C - Pre_D | 0.062 | 0.475 | 0.475 |
| Endocrine system | 0.026 | Pre_C - Pre_D | 2.233 | 0.013 | 0.013 |
| Environmental adaptation | 0.495 | Pre_C - Pre_D | 0.682 | 0.248 | 0.248 |
| Excretory system | 0.457 | Pre_C - Pre_D | 0.744 | 0.228 | 0.228 |
| Immune system | 0.620 | Pre_C - Pre_D | 0.496 | 0.310 | 0.310 |
| Nervous system | 0.107 | Pre_C - Pre_D | 1.612 | 0.053 | 0.053 |

## 2.5. Table S5

Table S5a Differential abundance results at the phylum level at baseline (DESeq2)

| Features | baseMean | log2FoldChange | lfcSE | stat | pvalue | padj | LOG10(pvalue) | LOG10(padj) |
| --- | --- | --- | --- | --- | --- | --- | --- | --- |
| Actinomycetota | 651.088 | -0.898 | 0.672 | -1.337 | 0.181 | 0.406 | 0.742 | 0.392 |
| Bacteroidota | 3.047 | -0.736 | 0.581 | -1.268 | 0.205 | 0.406 | 0.689 | 0.392 |
| Patescibacteria | 12.649 | 1.093 | 0.728 | 1.501 | 0.133 | 0.406 | 0.875 | 0.392 |
| Pseudomonadota | 18.461 | 0.684 | 0.620 | 1.102 | 0.270 | 0.406 | 0.568 | 0.392 |
| Synergistota | 8.582 | 0.981 | 0.852 | 1.152 | 0.249 | 0.406 | 0.603 | 0.392 |
| Verrucomicrobiota | 3.093 | -0.857 | 0.621 | -1.381 | 0.167 | 0.406 | 0.777 | 0.392 |
| Bacillota | 6157.468 | 0.125 | 0.292 | 0.427 | 0.669 | 0.753 | 0.174 | 0.123 |
| Campylobacterota | 2.893 | -0.351 | 0.654 | -0.537 | 0.591 | 0.753 | 0.228 | 0.123 |
| Fusobacteriota | 227.639 | 0.090 | 1.003 | 0.090 | 0.928 | 0.928 | 0.032 | 0.032 |

Table S5b Differential abundance results at the family level at baseline (DESeq2)

| Features | baseMean | log2FoldChange | lfcSE | stat | pvalue | padj | LOG10(pvalue) | LOG10(padj) |
| --- | --- | --- | --- | --- | --- | --- | --- | --- |
| Micrococcaceae | 15.525 | 2.049 | 0.651 | 3.149 | 0.002 | 0.024 | 2.785 | 1.624 |
| unclassified | 11.919 | 2.231 | 0.690 | 3.233 | 0.001 | 0.024 | 2.912 | 1.624 |
| Leptotrichiaceae | 8.461 | 2.186 | 0.808 | 2.705 | 0.007 | 0.066 | 2.166 | 1.181 |
| Bifidobacteriaceae | 55.625 | -1.892 | 0.933 | -2.028 | 0.043 | 0.251 | 1.371 | 0.600 |
| Bacteroidaceae | 3.073 | -1.289 | 0.638 | -2.021 | 0.043 | 0.251 | 1.364 | 0.600 |
| *Akkermansia*ceae | 3.137 | -1.226 | 0.647 | -1.896 | 0.058 | 0.280 | 1.237 | 0.553 |
| [Eubacterium]_coprostanoligenes_group | 60.666 | -1.505 | 0.939 | -1.602 | 0.109 | 0.452 | 0.962 | 0.345 |
| Coriobacteriaceae | 530.412 | -1.284 | 0.999 | -1.285 | 0.199 | 0.574 | 0.701 | 0.241 |
| Streptococcaceae | 5.320 | -0.632 | 0.599 | -1.056 | 0.291 | 0.574 | 0.536 | 0.241 |
| Clostridiaceae | 45.519 | 1.108 | 0.900 | 1.231 | 0.218 | 0.574 | 0.661 | 0.241 |
| Monoglobaceae | 26.897 | -0.886 | 0.849 | -1.043 | 0.297 | 0.574 | 0.528 | 0.241 |
| Ruminococcaceae | 1583.360 | 0.866 | 0.753 | 1.150 | 0.250 | 0.574 | 0.602 | 0.241 |
| Peptostreptococcaceae | 125.871 | 0.998 | 0.937 | 1.065 | 0.287 | 0.574 | 0.542 | 0.241 |
| Veillonellaceae | 10.249 | -0.905 | 0.702 | -1.290 | 0.197 | 0.574 | 0.706 | 0.241 |
| Saccharimonadaceae | 10.887 | 0.850 | 0.707 | 1.202 | 0.229 | 0.574 | 0.639 | 0.241 |
| Lachnospiraceae | 3888.037 | -0.370 | 0.383 | -0.966 | 0.334 | 0.605 | 0.476 | 0.218 |
| Lactobacillaceae | 2.784 | -0.486 | 0.601 | -0.810 | 0.418 | 0.674 | 0.379 | 0.172 |
| Enterobacteriaceae | 13.599 | 0.600 | 0.726 | 0.827 | 0.408 | 0.674 | 0.389 | 0.172 |
| Campylobacteraceae | 2.648 | -0.467 | 0.617 | -0.758 | 0.449 | 0.685 | 0.348 | 0.165 |
| Anaerovoracaceae | 6.915 | 0.428 | 0.638 | 0.670 | 0.503 | 0.729 | 0.299 | 0.137 |
| Oscillospiraceae | 141.478 | 0.551 | 0.883 | 0.624 | 0.533 | 0.736 | 0.274 | 0.133 |
| Pasteurellaceae | 3.211 | 0.326 | 0.631 | 0.517 | 0.605 | 0.798 | 0.218 | 0.098 |
| Butyricicoccaceae | 59.335 | -0.221 | 0.687 | -0.322 | 0.747 | 0.942 | 0.126 | 0.026 |
| Christensenellaceae | 6.898 | -0.103 | 0.778 | -0.133 | 0.895 | 0.963 | 0.048 | 0.016 |
| Eubacteriaceae | 2.143 | -0.094 | 0.593 | -0.158 | 0.874 | 0.963 | 0.058 | 0.016 |
| Fusobacteriaceae | 245.022 | -0.143 | 1.028 | -0.139 | 0.890 | 0.963 | 0.051 | 0.016 |
| Synergistaceae | 9.563 | 0.113 | 0.870 | 0.130 | 0.896 | 0.963 | 0.047 | 0.016 |
| Atopobiaceae | 11.089 | -0.019 | 0.793 | -0.025 | 0.980 | 0.980 | 0.009 | 0.009 |
| Eggerthellaceae | 31.291 | 0.027 | 0.734 | 0.037 | 0.971 | 0.980 | 0.013 | 0.009 |

Table S5c Differential abundance results at the species level at baseline (DESeq2)

| Features | baseMean | log2FoldChange | lfcSE | stat | pvalue | padj | LOG10(pvalue) | LOG10(padj) |
| --- | --- | --- | --- | --- | --- | --- | --- | --- |
| *Fusobacterium_periodonticum* | 11.387 | 3.391 | 0.766 | 4.427 | 0.000 | 0.000 | 5.020 | 3.698 |
| *Roseburia_hominis_A2_183* | 20.932 | 3.190 | 0.946 | 3.374 | 0.001 | 0.008 | 3.130 | 2.109 |
| *Eubacterium_ramulus* | 11.321 | (2.445) | 0.905 | (2.702) | 0.007 | 0.048 | 2.162 | 1.317 |
| *Anaerotignum_lactatifermentans* | 4.158 | (1.956) | 0.759 | (2.579) | 0.010 | 0.052 | 2.003 | 1.283 |
| *[Clostridium]_aldenense* | 4.718 | (1.364) | 0.636 | (2.143) | 0.032 | 0.135 | 1.494 | 0.870 |
| *Akkermansia_muciniphila* | 2.390 | (1.203) | 0.619 | (1.943) | 0.052 | 0.182 | 1.284 | 0.740 |
| *Eubacterium_sulci_ATCC_35585* | 3.400 | (0.970) | 0.602 | (1.613) | 0.107 | 0.320 | 0.972 | 0.495 |
| *Lachnospiraceae_bacterium_GAM79* | 8.022 | (0.952) | 0.728 | (1.307) | 0.191 | 0.401 | 0.719 | 0.396 |
| *Ruminococcus_torques_ATCC_27756* | 28.241 | 1.245 | 0.874 | 1.425 | 0.154 | 0.401 | 0.812 | 0.396 |
| *Ruminococcus_sp._N15.MGS_57* | 8.455 | 1.100 | 0.822 | 1.339 | 0.181 | 0.401 | 0.743 | 0.396 |
| *Oscillibacter_sp._PEA192* | 5.869 | 0.839 | 0.722 | 1.162 | 0.245 | 0.468 | 0.610 | 0.329 |
| *Streptomyces_sp._Q1* | 5.087 | (0.801) | 0.736 | (1.089) | 0.276 | 0.483 | 0.559 | 0.316 |
| *Rothia_mucilaginosa* | 2.491 | 0.450 | 0.522 | 0.862 | 0.389 | 0.583 | 0.410 | 0.234 |
| *Collinsella_tanakaei* | 14.382 | (0.937) | 1.051 | (0.892) | 0.372 | 0.583 | 0.429 | 0.234 |
| *Dorea_formicigenerans_ATCC_27755* | 12.212 | 0.576 | 0.796 | 0.723 | 0.469 | 0.654 | 0.328 | 0.184 |
| *Catonella_sp._oral_clone_AH153* | 3.916 | (0.384) | 0.674 | (0.570) | 0.569 | 0.654 | 0.245 | 0.184 |
| *Roseburia_inulinivorans* | 10.147 | 0.541 | 0.849 | 0.637 | 0.524 | 0.654 | 0.281 | 0.184 |
| *[Clostridium]_leptum* | 8.474 | (0.448) | 0.835 | (0.536) | 0.592 | 0.654 | 0.228 | 0.184 |
| unclassified | 7983.420 | (0.108) | 0.202 | (0.537) | 0.591 | 0.654 | 0.228 | 0.184 |
| *Anaerostipes_caccae_DSM_14662* | 2.760 | (0.180) | 0.539 | (0.335) | 0.738 | 0.775 | 0.132 | 0.111 |
| *[Clostridium]_scindens* | 5.731 | 0.073 | 0.737 | 0.099 | 0.921 | 0.921 | 0.036 | 0.036 |

## 2.6. Table S6

Table S6a Correlations between differential genera and KEGG level 2 functional modules (BH-adjusted)

| Genus | Feature | rho | P value | Q  value | stars | Genus |
| --- | --- | --- | --- | --- | --- | --- |
| *[Ruminococcus]_gnavus_group* | Immune disease | 0.712627 | 0.000136 | 0.042245 | * | *[Ruminococcus]_gnavus_group* |
| *[Ruminococcus]_gnavus_group* | Carbohydrate metabolism | 0.690882 | 0.000262 | 0.042245 | * | *[Ruminococcus]_gnavus_group* |

Table S6b Top 20 genus-functional module pairs ranked by absolute correlation coefficient

| Genus | Feature | rho | pvalue | qvalue | Genus |
| --- | --- | --- | --- | --- | --- |
| *[Ruminococcus]_gnavus_group* | Immune disease | 0.712627 | 0.000136 | 0.042245 | *[Ruminococcus]_gnavus_group* |
| *[Ruminococcus]_gnavus_group* | Carbohydrate metabolism | 0.690882 | 0.000262 | 0.042245 | *[Ruminococcus]_gnavus_group* |
| *[Ruminococcus]_gnavus_group* | Neurodegenerative disease | 0.660242 | 0.000607 | 0.065146 | *[Ruminococcus]_gnavus_group* |
| *Extibacter* | Nervous system | -0.64209 | 0.000956 | 0.069835 | *Extibacter* |
| *Extibacter* | Immune disease | 0.631856 | 0.00122 | 0.069835 | *Extibacter* |
| *[Eubacterium]_eligens_group* | Membrane transport | -0.61967 | 0.001613 | 0.069835 | *[Eubacterium]_eligens_group* |
| *Extibacter* | Endocrine system | -0.61523 | 0.001781 | 0.069835 | *Extibacter* |
| *[Eubacterium]_eligens_group* | Replication and repair | 0.613998 | 0.00183 | 0.069835 | *[Eubacterium]_eligens_group* |
| *[Eubacterium]_eligens_group* | Carbohydrate metabolism | -0.6109 | 0.001958 | 0.069835 | *[Eubacterium]_eligens_group* |
| *Faecalimonas* | Endocrine and metabolic disease | -0.60619 | 0.002169 | 0.069835 | *Faecalimonas* |
| *[Ruminococcus]_gnavus_group* | Endocrine and metabolic disease | -0.59056 | 0.003009 | 0.088068 | *[Ruminococcus]_gnavus_group* |
| *Faecalimonas* | Translation | -0.56747 | 0.00474 | 0.121431 | *Faecalimonas* |
| *UCG_002* | Transcription | 0.565699 | 0.004902 | 0.121431 | *UCG_002* |
| *Extibacter* | Neurodegenerative disease | 0.55895 | 0.005562 | 0.12792 | *Extibacter* |
| *Extibacter* | Endocrine and metabolic disease | -0.54616 | 0.007015 | 0.150584 | *Extibacter* |
| *Faecalimonas* | Folding, sorting and degradation | -0.53895 | 0.007964 | 0.160283 | *Faecalimonas* |
| *Extibacter* | Cellular community - prokaryotes | 0.528252 | 0.009567 | 0.181214 | *Extibacter* |
| *Hungatella* | Cell motility | 0.522749 | 0.01049 | 0.18766 | *Hungatella* |
| *Faecalimonas* | Replication and repair | -0.51653 | 0.01162 | 0.189417 | *Faecalimonas* |
| *[Ruminococcus]_gnavus_group* | Membrane transport | 0.512973 | 0.012311 | 0.189417 | *[Ruminococcus]_gnavus_group* |

## 2.7. Table S7

Table S7 Quality-control data for baseline relative abundance of Extibacter (DCP values reported as >300000 were truncated to 300000 for statistical analysis)

| SampleID | Group | mRECIST | AFP | DCP | Age | ECOG | *Extibacter* | log10_AFP | log10_*Extibacter* |
| --- | --- | --- | --- | --- | --- | --- | --- | --- | --- |
| A01 | C | PR | 15.14 | 7308 | 59 | 0 | 0 | 1.207904 | -6 |
| A03 | C | CR | 94145 | 58747 | 61 | 0 | 0 | 4.973802 | -6 |
| A04 | C | PR | 1399 | 2758 | 56 | 1 | 0 | 3.146128 | -6 |
| A05 | C | PR | 3603 | 26899 | 43 | 1 | 0 | 3.556785 | -6 |
| A08 | C | CR | 8310 | 113 | 58 | 0 | 0 | 3.919653 | -6 |
| A13 | C | CR | 8944 | 1056 | 49 | 0 | 0 | 3.95158 | -6 |
| A16 | C | PR | 25.9 | 5490 | 45 | 0 | 0 | 1.429752 | -6 |
| A17 | C | PR | 9.3 | 8510 | 67 | 1 | 0 | 1.012837 | -6 |
| A19 | C | PR | 9605 | 21009 | 46 | 0 | 0 | 3.982543 | -6 |
| A20 | C | PR | 2.24 | 20744 | 39 | 2 | 0 | 0.510545 | -6 |
| A02 | D | SD | 8512 | 53041 | 47 | 1 | 0 | 3.930083 | -6 |
| A06 | D | SD | 100 | 43203 | 41 | 1 | 0.155491 | 2.004321 | -0.80829 |
| A07 | D | SD | 48.36 | 3375 | 68 | 0 | 0.100525 | 1.693375 | -0.99772 |
| A09 | D | SD | 69132 | 72528 | 45 | 1 | 0 | 4.839685 | -6 |
| A10 | D | SD | 135 | 23180 | 54 | 1 | 0 | 2.133539 | -6 |
| A11 | D | SD | 151527 | 44150 | 51 | 1 | 0.001001 | 5.180493 | -2.99898 |
| A12 | D | SD | 2906 | 6134 | 56 | 1 | 0 | 3.463445 | -6 |
| A14 | D | SD | 541 | 7087 | 53 | 1 | 0.060317 | 2.733999 | -1.21955 |
| A15 | D | SD | 15213 | 17162 | 66 | 1 | 0.000278 | 4.182243 | -3.55401 |
| A18 | D | SD | 7637 | 300000 | 43 | 1 | 0 | 3.88298 | -6 |
| A21 | D | SD | 1016 | 2739 | 53 | 1 | 0.033721 | 3.007321 | -1.47209 |
| A22 | D | SD | 12450 | >300000 | 59 | 1 | 0 | 4.095204 | -6 |
| A23 | D | SD | 5 | 14282 | 48 | 1 | 0 | 0.778151 | -6 |

## 2.8. Table S8

Table S8 PERMANOVA results for paired gut microbiota beta diversity before and after combination therapy

| Metric | Group 1 | Group 2 | Sample size | Permutations | pseudo-F | P  value | Q  value |
| --- | --- | --- | --- | --- | --- | --- | --- |
| Bray Curtis | Post_treatment | Pre_treatment | 46 | 999 | 0.670281212 | 0.935 | 0.935 |
| weighted UniFrac | Post_treatment | Pre_treatment | 46 | 999 | 0.673688738 | 0.742 | 0.742 |
| unweighted UniFrac | Post_treatment | Pre_treatment | 46 | 999 | 0.854668702 | 0.805 | 0.805 |

## 2.9. Table S9

Table S9 PERMANOVA results for post-treatment gut microbiota beta diversity

| Metric | Group 1 | Group 2 | Sample size | Permutations | pseudo-F | p  value | q  value |
| --- | --- | --- | --- | --- | --- | --- | --- |
| **Bray-Curtis** | C | D | 23 | 999 | 1.1457066 | 0.232 | 0.232 |
| **Weighted UniFrac** | C | D | 23 | 999 | 1.0186171 | 0.448 | 0.448 |
| **Unweighted UniFrac** | C | D | 23 | 999 | 0.9823636 | 0.479 | 0.479 |

## 2.10.  Table S10

Supplementary Table S10. Baseline sensitivity analysis after excluding the lowest-depth sample A05

| Analysis category | Index / feature | Primary analysis P value | Sensitivity analysis excluding A05 P value | Primary result | Sensitivity result excluding A05 |
| --- | --- | --- | --- | --- | --- |
| Alpha diversity | Shannon index | 0.6869 | 0.9468 | Responder median = 3.266; non-responder median = 2.905 | Responder median = 3.260; non-responder median = 2.905 |
| Alpha diversity | Simpson index | 0.9259 | 0.7385 | Responder median = 0.886; non-responder median = 0.893 | Responder median = 0.862; non-responder median = 0.893 |
| Alpha diversity | Observed features | 0.0673 | 0.1089 | Responder median = 175; non-responder median = 134 | Responder median = 171; non-responder median = 134 |
| Beta diversity | Bray-Curtis PERMANOVA | 0.7600 | 0.7280 | R² = 0.039; F = 0.850 | R² = 0.041; F = 0.853 |
| Differential genus | Extibacter | 0.0179 | 0.0242 | Detected in 0/10 responders and 6/13 non-responders | Detected in 0/9 responders and 6/13 non-responders |

## 2.11.  Table S11

Table S11. Bootstrap-based internal assessment of exploratory ROC models

| Model | Apparent_AUC | Bootstrap_mean_AUC | Bootstrap_median_AUC | Bootstrap_95CI_lower | Bootstrap_95CI_upper | Valid_bootstrap_runs |
| --- | --- | --- | --- | --- | --- | --- |
| Extibacter alone | 0.731 | 0.729 | 0.731 | 0.607 | 0.861 | 1000 |
| Extibacter + AFP | 0.777 | 0.801 | 0.802 | 0.646 | 0.946 | 1000 |

Note: Apparent AUCs were calculated using the original baseline cohort. Bootstrap mean and median AUCs, together with percentile-based 95% confidence intervals, were estimated from 1,000 bootstrap resampling iterations. These analyses were performed as internal stability assessments and should not be interpreted as external validation.
